# Multiplexed cytokine profiling identifies diagnostic signatures for latent tuberculosis and reactivation risk stratification

**DOI:** 10.1371/journal.pone.0316648

**Published:** 2025-04-09

**Authors:** Krista Meserve, Cole A. Chapman, Mingrui Xu, Haowen Zhou, Heather M. Robison, Heather R. Hilgart, Pedro P. Arias-Sanchez, Balaji Pathakumari, Manik R. Reddy, Kale A. Daniel, Thomas M. Cox, Courtney L. Erskine, Paige K. Marty, Mounika Vadiyala, Snigdha Karnakoti, Virginia Van Keulen, Elitza Theel, Tobias Peikert, Colleen Bushell, Michael Welge, Rafael Laniado-Laborin, Ruoqing Zhu, Patricio Escalante, Ryan C. Bailey

**Affiliations:** 1 Department of Chemistry, University of Michigan, Ann Arbor, Michigan, United States of America; 2 Department of Statistics, University of Illinois Urbana-Champaign, Champaign, Illinois, United States of America; 3 Department of Statistics, University of Virginia, Charlottesville, Virginia, United States of America; 4 Department of Laboratory Medicine, Mayo Clinic, Rochester, Minnesota, United States of America; 5 Department of Medicine, Division of Pulmonary and Critical Care Medicine, Mayo Clinic, Rochester, Minnesota, United States of America; 6 Department of Immunology, Mayo Clinic, Rochester, Minnesota, United States of America; 7 National Center for Supercomputing Applications, University of Illinois Urbana-Champaign, Urbana, Illinois, United States of America; 8 Clinica y Laboratorio de Tuberculosis, Facultad de Medicina y Psicologia, Hospital General Tijuana, Universidad Autonoma de Baja California, ISESALUD, Tijuana, Baja California, Mexico; Showa University Fujigaoka Hospital, JAPAN

## Abstract

Active tuberculosis (TB) is caused by *Mycobacterium tuberculosis* (Mtb) bacteria and is characterized by multiple phases of infection, leading to difficulty in diagnosing and treating infected individuals. Patients with latent tuberculosis infection (LTBI) can reactivate to the active phase of infection following perturbation of the dynamic bacterial and immunological equilibrium, which can potentially lead to further Mtb transmission. However, current diagnostics often lack specificity for LTBI and do not inform on TB reactivation risk. We hypothesized that immune profiling readily available QuantiFERON-TB Gold Plus (QFT) plasma supernatant samples could improve LTBI diagnostics and infer risk of TB reactivation. We applied a whispering gallery mode, silicon photonic microring resonator biosensor platform to simultaneously quantify thirteen host proteins in QFT-stimulated plasma samples. Using machine learning algorithms, the biomarker concentrations were used to classify patients into relevant clinical bins for LTBI diagnosis or TB reactivation risk based on clinical evaluation at the time of sample collection. We report accuracies of over 90% for stratifying LTBI + from LTBI– patients and accuracies reaching over 80% for classifying LTBI + patients as being at high or low risk of reactivation. Our results suggest a strong reliance on a subset of biomarkers from the multiplexed assay, specifically IP-10 for LTBI classification and IL-10 and IL-2 for TB reactivation risk assessment. Taken together, this work introduces a 45-minute, multiplexed biomarker assay into the current TB diagnostic workflow and provides a single method capable of classifying patients by LTBI status and TB reactivation risk, which has the potential to improve diagnostic evaluations, personalize treatment and management plans, and optimize targeted preventive strategies in Mtb infections.

## Introduction

Active tuberculosis (TB) is a complex disease resulting from infection with *Mycobacterium tuberculosis* (Mtb), which can have pulmonary and/or extrapulmonary involvement. TB infection is estimated to be present in around 25% of the world’s population and continues to be a fatal disease, with an attributed 10.6 million cases and 1.3 million deaths worldwide in 2022 [1,2]. TB disease is currently regarded as a continuum of various phases of infection, with each phase requiring different methods of diagnosis and treatment regimes. This adds to the complex task of global TB eradication [3–5]. Upon infection, the host immune response attempts to contain and clear the Mtb, but under many circumstances only succeeds in sequestering the bacteria in granulomas found in different stages of maturation primarily in the lungs and lymph nodes [6–8]. Granuloma development aids Mtb persistence by isolating the bacteria from the host’s immune response, but also protects the host from continued bacterial growth and replication. The dynamic bacterial and immunological equilibrium induced by the granulomatous immune response results in a phase of TB infection called latent TB infection (LTBI), in which the host is asymptomatic with no indications of active disease on radiographs [8]. It is estimated that from all Mtb-infected individuals worldwide, 20% manifest clinically as TB disease and 80% persist as LTBI, totaling an estimated 2 billion people [6,9,10].

Approximately 90% of LTBI patients who are immunocompetent will stay asymptomatic and non-contagious [4,6]. The remaining patients will eventually reactivate from LTBI to active TB through a disruption in the dynamic equilibrium resulting in rupture of the protective granuloma and immune response. The Mtb, no longer held in a quiescent state, will then begin to replicate usually within the lungs and lymph node compartments of the host. These infection reactivation and disease progression events facilitate further transmission of Mtb. Many TB reactivation events occur within 24 months of initial infection, highlighting the importance of diagnosing the early latent phase [4]. Therefore, a key to eradicating TB involves both the identification of individuals with LTBI and the stratification of patients according to their risk of reactivation from LTBI to active TB, since preventative antibiotic therapy would be most beneficial for high-risk individuals.

To reach this ambitious goal, high performance diagnostics for accurate LTBI diagnosis and risk stratification are urgently needed. There currently is no stand-alone gold standard test for LTBI. Diagnosis is typically achieved via positive immune responses to Mtb in an interferon-gamma release assay (IGRA) and/or tuberculin skin test (TST), in conjunction with the absence of active TB markers in clinical-radiological examinations. The TST is a less specific test compared to IGRAs, since immune responses to the purified protein derivative utilized are cross-reactive against other mycobacterial and Bacillus Calmette-Guérin (BCG) vaccine antigens.

One of the available IGRAs is the QuantiFERON-TB Gold Plus test (QFT), which identifies individuals who have been infected with or exposed to Mtb. The QFT assay involves stimulation of lymphocytes in whole-blood samples with Mtb-specific antigens and controls followed by quantitation of interferon-gamma (IFN-γ) in the separated plasma by using an enzyme-linked immunosorbent assay (ELISA) or chemiluminescence immunoassay (CLIA) [11,12]. IGRAs have limitations when applied to LTBI diagnostics, including indeterminate test results in immunosuppressed patients, specifically those with altered IFN-γ host responses. IGRAs are also unable to differentiate between LTBI and active TB and have less than 3% predictive value in determining risk of TB reactivation over time [5,13–16]. Recent research into determining individuals at very high risk of TB reactivation (incipient TB) has focused on whole-blood gene expression or ribonucleic acid (RNA) signatures, which can yield false positive results with viral infections, are generally less stable than protein biomarkers, and require RNase-free sample processing [17–21]. Therefore, there is a need for improved protein-based biomarker signatures for both LTBI diagnosis and TB reactivation risk stratification.

Our work is motivated by the World Health Organization’s goal of decreasing the annual TB incidence rate by 80% before 2030. We aim to expand the TB diagnostic toolbox with a multiplexed immunoassay tool that addresses both LTBI status and risk of TB reactivation using a sample input of readily available stimulated QFT plasma. Multiplexed assays are an area of intense interest in clinical diagnostics, as the complex biological processes of the immune system are more informatively understood using multi-biomarker profiles compared to any single biomarker [22–27]. In a TB context, multiplexed assays can identify host biomarkers beyond IFN-γ as indicators of Mtb infection phase, potentially reducing false negatives in patients with IFN-γ dysregulation or due to IFN-γ independent immune responses against Mtb [26,28]. There has been a significant focus on developing multi-biomarker signatures for determining active TB status [29–35] and some studies have incorporated multi-biomarker signatures for other phases of TB infection, such as LTBI [36–40]. These studies demonstrate promise for using cytokine biomarkers to differentiate various phases of TB infection. However, many do not address variability in immunological response between patients and none feature applicability towards combining both LTBI determination and TB reactivation risk stratification in the same assay at initial assessment.

This work utilizes a silicon photonic microring resonator biosensor platform capable of rapid, multiplexed biomarker detection to quantify host proteins in QFT-stimulated plasma samples. The biomarker concentrations are used to classify patients into relevant clinical bins for LTBI diagnosis or TB reactivation risk inference using machine learning algorithms. The 45-minute assay with automated fluid handling is faster and requires less manual manipulation than traditional ELISA assays and, when coupled with machine learning, provides a method to identify important diagnostic biomarkers for LTBI and TB reactivation risk classifications. We have previously applied this multiplexed microring platform in various clinical capacities, including LTBI-relevant diagnostics [41–47]. However, these studies utilized samples emanating from *in vitro* stimulation of peripheral blood mononuclear cells (PBMCs) with six different antigens or controls, which is technically laborious and time consuming. Notably, though, this work demonstrated the applicability of the platform coupled with machine learning methods to generate biomarker profiles that were not only biologically driven, but also led to predictive accuracies for LTBI and high-risk status that suggested a strong potential for clinical diagnostics. We now postulate that employing this multiplexed assay workflow and bioinformatic analysis with readily available residual QFT plasma will create a more rapid profiling approach directly amenable to the current clinical workflow for TB diagnostics. The goal of this work is to determine the diagnostic accuracy of our method to diagnose LTBI and identify individuals with increased risk (high risk) and reduced risk (low risk) of TB reactivation.

Herein, we report predictive accuracies for classifying LTBI positive individuals from a population of patients who received QFT testing at the Mayo Clinic using both absolute and precision normalized biomarker concentrations. We further stratify the LTBI positive patient group into high- or low-risk of reactivation and report predictive accuracies for classifying both risk designations. Further, we identify the most important biomarker variables for all the models and highlight a dependence on six of the thirteen biomarkers in the panel. Through this implementation, we demonstrate how our microring resonator platform is amenable to the current TB diagnostic pipeline and can increase the amount of clinical information gleaned from a single test. This work enables improvement in LTBI diagnosis and has the potential to optimize individual preventive treatment and management decisions for subjects exposed to Mtb.

## Methods

### Subject enrollment and clinical designations

This study was approved by the Mayo Clinic Institutional Review Board (IRB #09–003253) and Olmsted County Public Health Services. All study participants signed an informed written consent. Subjects were enrolled at the Mayo Clinic in Rochester, Minnesota, between August 3, 2017 and December 6, 2023. Risk factors for TB infection, TB progression, and/or TB reactivation were obtained through a questionnaire and review of medical records as previously described [48,49]. LTBI diagnoses were made based on the Centers for Disease Control and Prevention (CDC) current guidelines criteria, TB risk factors, and prior TST and QuantiFERON-TB Gold In-Tube or Gold In-Tube Plus results (Qiagen, Germantown, MD) [49,50]. The study subjects included TB unexposed individuals with negative IGRA testing results (LTBI negative cases), and subjects with LTBI at varying risk for developing active TB infection, including untreated LTBI patients and patients who had received treatment for LTBI. A modified multifactorial predictive modeling platform (Online TST/IGRA interpreter) was applied to estimate the cumulative risk of TB reactivation in all subjects [51,52]. The platform accounts for known risk factors for TB progression and reactivation, such as human immunodeficiency virus (HIV) infection, diabetes mellitus, treatment with TNF-α antagonists, and body weight, and is adjusted by LTBI treatment effect.

Three clinical designations were used in this study: LTBI status, high-risk LTBI status, and low-risk LTBI status. Together, these designations provide information relevant to TB reactivation risk assessments and, thus, individual treatment recommendations relevant for clinicians and public health providers who screen, diagnose, and manage LTBI patients who have a possible or confirmed exposure to an active pulmonary TB patient and who are at risk of TB reactivation. CDC guidelines for diagnosis of LTBI were used to classify subjects as LTBI positive (LTBI+) or negative (LTBI−) [50]. Subjects with a LTBI+ diagnosis were deemed at high risk of TB reactivation if they could be classified as either (1) individuals born in and/or from TB endemic areas having untreated LTBI with both TST+ and prior IGRA+ results, (2) having untreated LTBI with TST+ conversion, prior IGRA− results, and prior TB exposures, (3) having prior close TB exposure and subsequent IGRA+ results, (4) being immunosuppressed with untreated LTBI, or (5) having risk factors, such as TB exposure, IGRA+ results, and a cumulative risk of TB reactivation ≥  4.1% based on the adjusted TST/IGRA interpreter. Subjects with a LTBI+ diagnosis were further classified as low risk of TB reactivation if they were LTBI+, completed guidelines-based preventative treatment for LTBI, and had a low likelihood for subsequent TB exposures at the time of study enrollment, such as those living in low TB endemic areas.

### Sample collection and QuantiFERON testing

Three milliliters of blood were collected from each subject and sent for QFT testing at the Mayo Clinic’s clinical laboratories. Initial study participants (39/165 patients) were analyzed using the QuantiFERON®-TB Gold In-Tube (Qiagen) kit. The QFT assay was performed according to manufacturer recommendations that included three separate in-tube stimulation conditions: a negative control (NIL), positive control (MIT), and the Mtb-specific peptide mixture for CD4+ cell stimulation (AG) [51,53]. During the study, the Mayo Clinic laboratory transitioned to the QuantiFERON-TB Gold Plus (Qiagen) kit [54]. This updated kit contained the stimulation categories of NIL, MIT, and Mtb-specific peptide mixture (now termed TB1 and shown to be akin to the AG stimulation in the original QFT assay). The updated kit included an additional Mtb-specific stimulation, TB2, that contained the same peptide cocktail as TB1 along with proprietary peptides to elicit both CD4+ and CD8+ T-cell responses [55]. For the algorithms developed in this work, biomarker profiling data from the TB2 tube was not used since the NIL, MIT, and AG/TB1 stimulations are ubiquitous for both QFT assays used across all patients in the study.

Patient blood was stimulated in-tube and the plasma was separated from the red blood cells and analyzed for IFN- γ concentration by an ELISA or CLIA. The results were interpreted as per the kit instructions. While TB2 tubes were excluded from biomarker profiling for continuity to the original QFT assay, the tubes were still used in clinical designation after transitioning to the updated QFT assay, as per Qiagen instructions. In the original QFT assay, a positive result was declared if measured levels of IFN-γ included NIL ≤ 8.0 IU/mL, the IFN- γ concentration difference between TB1 and NIL tubes (TB1-NIL) ≥ 0.35 IU/mL, and TB1-NIL ≥  25% of the NIL IFN-γ value. In the updated QFT assay, a positive result was declared if IFN-γ concentrations in NIL ≤ 8.0 IU/mL, TB1-NIL or TB2-NIL ≥ 0.35 IU/mL, and TB1-NIL or TB2-NIL ≥  25% of the NIL IFN-γ value. Samples that were declared indeterminant were excluded from the study. Leftover plasma from each QFT tube was stored at -80°C after testing and thawed immediately before multiplexed cytokine analyses.

### Multiplexed biomarker detection

Silicon photonic microring resonators were used to perform multiplexed assays for quantifying thirteen protein biomarkers in this report. Briefly, changes in the local refractive index at the surface of each sensor causes a shift in the resonant wavelength of light supported by each microring structure. Capture antibodies specific to the target proteins were arrayed across the sensor chip surface such that there were four technical replicates per target. The sensing scheme resembled a sandwich-style ELISA, with the immobilized capture antibody, target protein, and biotinylated tracer antibody forming a highly specific sandwich complex that was tagged with streptavidin-conjugated horse-radish peroxidase (SA-HRP) [56–58]. The final assay step included an amplification reagent (4-chloro-1-napthol) that was catalytically converted to an insoluble product by the HRP, resulting in local deposition of a precipitate onto the microring that in turn generated a greater shift in resonant wavelength proportional to the target analyte concentration. Multiplexed calibration curves (S1 Fig in S2 File) were used to convert the resulting shifts in resonant wavelength to concentrations for each of the thirteen biomarkers in the QFT plasma samples. The technology provided automated, simultaneous analysis of two samples with near real-time output response. The optimized assay featured a time-to-result of 45 minutes, required less than 250 μL of QFT plasma sample, and provided limits of detection in the picogram per milliliter range. A detailed description of the technology, sensing principles, instrumentation, sensor chip preparation, assay parameters, and calibration construction can be found in the Supplemental Information.

### Assay panel and patient sample analysis

The 13-plex cytokine and chemokine panel deployed in this study was selected based on previously published work [46,47] and consisted of IL-1β [59], IL-2 [60], IL-6 [61], IL-10 [62], IL-15 [63], IL-17 [64], CCL2/MCP-1 [65], CCL3/MIP-1α [66], CCL4/MIP-1β [67], CCL8/MCP-2 [68], IFN- γ [69], IP-10 [70,71], and TNF-⍺[72]. The antibody pairs and standard proteins for each of these targets are detailed in S1 Table in S2 File. Each patient sample was analyzed simultaneously on a single sensor chip at two dilutions in under 45 minutes using residual QFT stimulated plasma (example sample data output shown in S2 Fig in S2 File). Sample dilutions of 10X and 2X were chosen to allow coverage of the necessary dynamic ranges for all cytokines in the multiplexed panel. Each plasma sample was removed from −80°C, thawed at 4°C, and diluted two- and ten-fold in 1X phosphate-buffered saline containing 0.5% bovine serum albumin to a total volume of 350 μL. The instrumental method for sample analysis is described in the Supplemental Information. Institutionally approved biohazard safety level 2 precautions were exercised while handling the QFT plasma samples.

Microring data analysis was completed as described previously [73]. The relative shift before the amplification step was subtracted from the relative shift at the end of the final buffer rinse to determine a final net shift for each of the thirteen targets per sample across both dilutions. Using the calibration curves, the net shifts for both analyzed dilutions (10X and 2X) were converted to analyte concentration using the corresponding calibration (10% plasma and 50% plasma, respectively). The technical replicates (n = 4) were averaged for each target. The dilution that resulted in a concentration closest to the inflection point of the specific target’s calibration curve was selected, corrected for dilution factor, and used for further analysis. Data points that exceeded the saturating point of a target’s specific calibration were assigned a value ten times greater than the highest measurable concentration, and data points below the specific target’s limit of detection were assigned as zero. The analysis workflow yielded one concentration value for each of the thirteen biomarkers across the three QFT simulated plasma samples, resulting in 39 absolute biomarker concentrations per patient for analysis.

### Precision normalization

We normalized biomarker levels within the various QFT samples for each patient to determine if accounting for patient-to-patient variation in baseline immunity would result in more accurate models. For each individual biomarker, the normalization procedure included subtracting the negative control concentration from that of the Mtb-specific stimulation to correct for basal cytokine immune response (TB1-NIL), subtracting the positive control concentration from that of the Mtb-specific stimulation to assess TB-specific response against mitogenic response (TB1-MIT), and subtracting the positive control concentration from that of the negative control as a measure of functional (inducible) immune response of the subject (MIT-NIL). Taken together, the three normalized conditions calculated for each of the 13 measured biomarker concentrations led to 39 variables per patient for analysis.

### Statistical testing

Comparing the biomarker concentrations between clinical designations was completed using two-sample Wilcoxon rank-sum tests, also known as Mann-Whitney U tests or Wilcoxon-Mann-Whitney (WMW) tests. A WMW test is a non-parametric statistical hypothesis test that quantifies differences in the distribution of data between two populations [74,75]. WMW tests were chosen for comparing the biomarkers between clinical designations because the samples between groups meet the independent, ordinal, and continuous test assumptions, intervals may not be constant, outliers are present, and this test is suited for these sample sizes. The null hypothesis is that the distribution of the groups being compared are identical, with the alternative hypothesis being that the distribution of the groups is not identical. The absolute cytokine concentrations were log-transformed for data visualization.

### Random forest machine learning

This study employs the random forest approach to classify individuals into LTBI and TB reactivation risk clinical designations based on collected biomarker features [76]. Random forest is an ensemble learning method that constructs and aggregates a multitude of decision trees to determine a classification outcome and can detect nonlinear effects of covariate features. The aggregation of many trees increases flexibility and improves accuracy when compared to using a single decision tree, while feature selection ranks a feature’s effect on the classification of a clinical designation, identifying the most important variables for classification [77]. All informatics and data plotting were performed using R version 4.3.1.

The specifics of the random forest algorithm, variable selection, and informatic handling of repeated entries are described in the Supplemental Information. The visual representation of random forest outputs are receiver operator characteristic (ROC) curves [78]. ROC curves graphically depict the trade-off between the true positive rate and false positive rate across various classification thresholds. They can be compared using the area under the curve (AUC), a metric of the model’s ability to discriminate between clinical classification bins. An AUC of one indicates 100% sensitivity and specificity across all thresholds, while an AUC of 0.5 is equivalent to random chance. All ROC curves in our analysis are calculated based on the out-of-bag predictions from random forests [76]. Out-of-bag predictions mimic the leave-one-out mechanism used for evaluating a machine learning model and are less vulnerable to overfitting.

With a dataset comprising 78 possible biomarker features for each subject, the presence of numerous potentially non-informative variables introduces non-robustness and variance into our final label estimation. Our classification pipeline was comprised of two main parts: (1) biomarker feature importance evaluation and (2) variable selection and classification model construction. While there are many existing variable selection methods [79–81], random forest provides a sensible and robust metric, termed variable importance, to gauge each variable’s contribution to prediction accuracy [76]. Empirical confidence intervals can be constructed by leveraging variable importance scores from each tree, which ensures robustness in importance score estimation [82]. Variables with high importance rankings, identified under a predetermined threshold, were integrated into a refined (reduced) random forest model for further training. Classification performance evaluation, AUC, was carried out using out-of-bag samples containing the selected informative variables.

A key challenge in this study arose from repeated records, or multiple samples at different time points from a unique subject, within the dataset. This could lead to correlations between in-bag and out-of-bag data and result in potential biases in prediction accuracy estimates. To mitigate this issue, we proposed a modification to the random forest algorithm. Initially, a single datum was sampled for each individual tree to ensure uniqueness. Subsequently, within the bagging process, repeated observations were managed to ensure proper assignment to in-bag or out-of-bag samples, thereby impeding the robustness of prediction accuracy estimates. Further details are provided in the Supplementary Information.

## Results

### Patient demographics

This study enrolled 144 individual patients, and all provided a blood draw for QFT testing (Table 1). Seventeen of the patients provided a later second time point and four provided a third time point as part of an ongoing longitudinal profiling project, leading to a total of 165 sample sets included in our analysis. Each set of QFT samples was analyzed independently, and the machine learning algorithm accounted for repeat measurements to minimize correlation bias. Of the 165 samples, 78 came from LTBI+ individuals. LTBI+ subjects were younger and more likely to be male, non-Caucasian, and born outside the United States than the LTBI− subjects. There was no statistical difference in the frequency of BCG vaccination and immunosuppression across these two groups. Within the LTBI+ study population, 50 samples were obtained from individuals designated to be at high reactivation risk and 25 were derived from persons designated as being at low reactivation risk, with the remaining three being indeterminant risk. The high-risk patients were more likely to be male, but no other demographic data was significantly different between these study groups. As expected, the treatment-adjusted predicted cumulative TB risk and annual TB risks, as well as the QFT (TB1 – NIL) IFN-γ concentrations, were statistically lower in the low-risk group compared with the high-risk study group (Table 1).

**Table 1:** Patient demographic data for the 165 samples in the patient cohort.

Subjects, no. (%)							
	All^a^ (n=165)	LTBI− (n= 84)	LTBI+ (n= 78)	*P* value^b^	High Risk+ (n=50)	Low Risk+ (n=25)	*P* value^c^
Sex (female)	101 (61.2)	59 (70.2)	42 (53.8)	0.031	22 (44)	17 (68)	0.050
Age, years							
Mean ± SD	52.32 ± 17.8	57.2 ± 17.4	47.3 ± 16.8	<0.001	46.5 ± 16.7	50.8 ± 17.1	0.331
Range	19 – 84	19 – 84	22 – 81		22 – 81	25 – 78	
Ethnicity							
Caucasian	117 (70.9)	73 (86.9)	43 (55.1)	<0.001	25 (50)	17 (68)	0.379
African American	11 (6.7)	2 (2.4)	8 (10.3)		6 (12)	1 (4)	
Asian Pacific	18 (10.9)	8 (9.5)	10 (12.8)		8 (16)	1 (4)	
Hispanic	7 (4.2)	1 (1.2)	5 (6.4)		3 (6)	2 (8)	
Others	12 (7.3)	0 (0)	12 (15.4)		8 (16)	4 (16)	
Place of Birth^d^							
United States Born	111 (67.3)	70 (83.3)	40 (51.3)	<0.001	24 (48)	15 (60)	0.489
Foreign Born (High TB)	5 (3)	3 (3.6)	2 (2.6)		1 (2)	1 (4)	
Foreign Born (Low TB)	49 (29.7)	11 (13.1)	36 (46.2)		25 (50)	9 (36)	
History of BCG vaccination							
Yes	89 (53.9)	50 (59.5)	37 (47.4)	0.063	18 (36)	16 (64)	0.120
No	25 (15.2)	11 (13.1)	14 (17.9)		12 (24)	2 (8)	
Unknown	29 (17.6)	9 (10.7)	19 (24.4)		14 (28)	5 (20)	
Probably no	22 (13.3)	14 (16.7)	8 (10.3)		6 (12)	2 (8)	
Occupation							
Health care worker, direct patient care	36 (21.8)	16 (19)	20 (25.6)	0.1361	13 (26)	7 (28)	0.975
Health care worker, no direct patient care	34 (20.6)	14 (16.7)	20 (25.6)		13 (26)	6 (24)	
Other	95 (57.6)	54 (64.3)	38 (48.8)		24 (48)	12 (48)	
Predicted cumulative TB risk (%)^e^							
Mean ± SD	4.23 ± 12.15	0.57 ± 1.75	8.24 ± 16.73	<0.001	8.88 ± 15.71	7.17 ± 19.78	0.045
Range	0 – 100	0 – 8.97	0 – 100		0 – 61.25	0 – 100	
Adjusted predicted cumulative TB risk (%)^f^							
Mean ± SD	2.93 ± 8.6	0.25 ± 0.73	5.8 ± 11.82	<0.001	7.87 ± 13.99	2.21 ± 5.05	<0.001
Range	0 – 61.25	0 – 3.81	0 – 61.25		0 – 61.25	0 - 25	
Adjusted predicted annual TB risk (%)^f^							
Mean ± SD	0.09 ± 0.27	0.01 ± 0.03	0.19 ± 0.36	<0.001	0.24 ± 0.41	0.11 ± 0.28	<0.001
Range	0 – 1.99	0 - 0.12	0 – 1.99		0 – 1.99	0 – 1.34	
History of immunosuppression (+) ^g^	21 (12.7)	10 (11.9)	11 (14.1)	0.230	5 (10)	5 (20)	0.286
Tuberculin skin test							
TST (+)	53 (32.1)	18 (21.4)	35 (44.9)	<0.001	24 (48)	11 (44)	0.285
TST mm induration	6.21 ± 8.22	3 ± 6.21	9.64 ± 8.91	0.041	12.88 ± 8.39	6.4 ± 8.79	0.127
Mean ± SD; range	0 – 20	0 – 15	0 – 20		0 – 20	0 – 17	
IGRA test							
QFT (+)	62 (37.6)	0 (0)	50 (76.9)	<0.001	41 (82)	16 (64)	0.064
QFT results (IU/mL)	1.59 ± 2.82	0.02 ± 0.06	3.29 ± 3.31	<0.001	4.33 ± 3.41	1.25 ± 1.92	<0.001
Mean ± SD; range^h^	−0.07 - 10	−0.07 – 0.23	−0.01 - 10		0 – 10	−0.1 – 8.03	

Abbreviations: IGRA =  Interferon-gamma release assay; SD =  Standard deviation; High TB =  High incidence of TB; Low TB =  Low incidence of TB; QFT =  QuantiFERON TB Gold In-Tube™ or QuantiFERON TB Gold Plus™; TST =  Tuberculin skin test. HIV =  Human immunodeficiency virus. HCW =  healthcare worker.

^a^The total sample set analyzed were 165 samples, encompassing 144 unique subjects, including 3 active TB patients, 13 subjects providing 2 separated samples, and 4 subjects providing 3 samples, separated by 5–7 months in testing, representing unique samples.

^b^P value for comparison between subjects with LTBI− vs. LTBI+ group designations (Mann–Whitney U test or r x c Exact contingency table).

^c^P value for comparison between subjects with High Risk+ group designation (including 3 patients with active TB) vs. Low Risk+ group designation (excluding 3 LTBI subjects with indeterminate Low risk designations). (Mann–Whitney U test or r x c Exact contingency table).

^d^High incidence of TB was defined as a country with ≥ 20 cases per 100,000 population per year. 2023 WHO Global tuberculosis report (http://www.who.int/tb/publications/global_report/en/).

^e^Estimates for the individual cumulative risk of TB reactivation are based on “The Online TST/IGRA interpreter” prediction modeling that includes TST and IGRA results, risk factors for LTBI and risk factors for progression to active TB ((http://www.tstin3d.com/en/calc.html) [51,52].

^f^Adjusted estimates for the individual and cumulative risk of TB reactivation are based on “The Online TST/IGRA interpreter” prediction modeling that includes TST and IGRA results, risk factors for LTBI and risk factors for progression to active TB (http://www.tstin3d.com/en/calc.html) as previously described [51,52].

^g^Study subjects included 21 patients with non-HIV immunosuppressed conditions.

^h^QFT results include Interferon-gamma levels (IU/mL) of antigen tube minus nil from QuantiFERON TB Gold In-Tube™ and TB1 tube from QuantiFERON TB Gold Plus™.

### Classification of LTBI positive subjects using absolute and normalized cytokine concentrations

**Fig 1:**
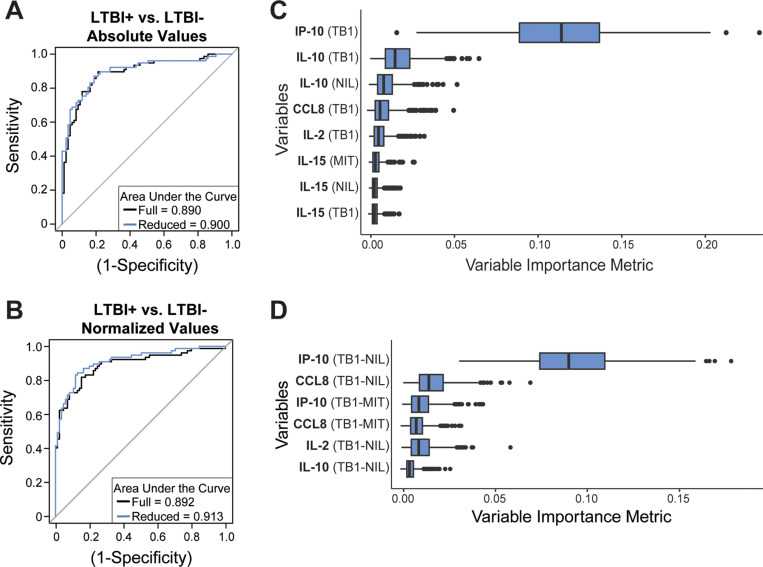
LTBI classification using absolute and normalized stimulated cytokine concentrations. Random forest classification is represented as receiver operator characteristic (ROC) curves when using absolute (A) and normalized (B) stimulated biomarker concentrations as the input variables. The most important variables identified through the variable importance metrics using the full set of absolute (C) and normalized (D) biomarker concentrations were used to generate the reduced data models. The variable importance metrics for all input variables in both models are presented in the Supplemental Information.

Using the 165 sample sets in our study, we first aimed to differentiate LTBI+ and LTBI− subjects using the random forest machine learning algorithm adjusted for repeated samples. Leave-one-out cross-validation was completed to determine the ability of the biomarker combinations to identify LTBI+ patients from all others in the study population. The absolute cytokine concentration values from the stimulated plasma samples resulted in a predictive accuracy (AUC) of 89.0% (Fig 1A) and the normalized cytokine values resulted in an accuracy of 89.2% (Fig 1B) using the full data sets of 39 absolute or normalized variables, respectively. Variable reduction was completed by using the variable importance metric from the full data set (S2 and S3 Tables in S2 File) to refine the model. This is done by restricting the model to variables that have the highest standardized importance (variable importance divided by its estimated standard deviation) and refit with the reduced set of variables. The reduced model utilizing eight (out of 39) variables (Fig 1C) achieved a prediction accuracy of 90.0% using absolute cytokine values, while the reduced model with a size of six (Fig 1D) resulted in a prediction accuracy of 91.3% using normalized cytokine values. The normalized cytokine value-derived model improved the classification accuracy by just 1.3% compared to the absolute cytokine value-derived model, indicating the normalized values did not substantially enhance differentiation of the LTBI+ patients from the remaining patients in the study population.

The response of IP-10 in Mtb-specific stimulations is the dominant predictive biomarker in both models. There is an overlap in the important biomarkers between models that include IP-10, CCL8, IL-2, and IL-10, which suggests the importance of these biomarkers regardless of which data type was used to generate the model. Five of the eight variables in the reduced absolute cytokine value-derived model are associated with the TB1 stimulated plasma, while all six in the reduced normalized cytokine value-derived model are associated with the TB1 stimulation. This indicates reliance on Mtb-specific peptide stimulations for making the LTBI classification designation. Variables associated with NIL and MIT stimulated plasma samples are still present in the absolute cytokine reduced models, albeit with lower importance metrics. This indicates that contributions from a subject’s functional immune response have value in classifying LTBI status. A key result to highlight is that the reduced models that achieved 90%+ accuracy were generated using different stimulations of just four (normalized) or five (absolute) biomarkers. These results suggest a dependence on a select few cytokines in the multiplexed profiling panel.

**Fig 2:**
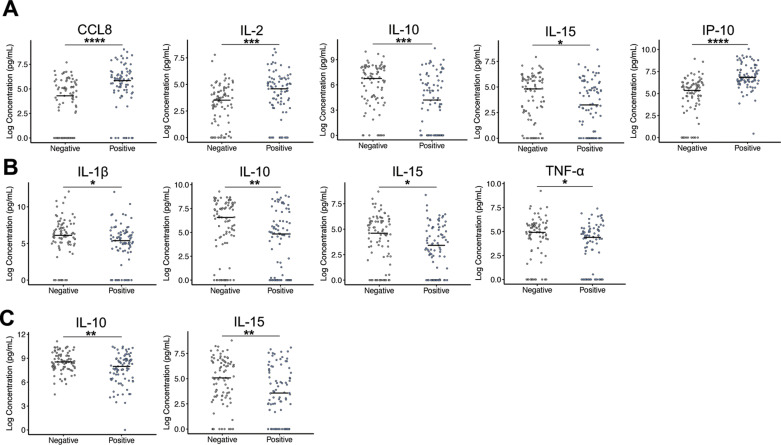
Significantly different distributions of absolute cytokine values between LTBI positive and negative patients. Target concentrations from stimulation conditions of TB1 (A), NIL (B), and MIT (C) that were significantly different between clinical populations of LTBI+ and LTBI− patients are shown. Median value of clinical population represented as a black line. Data visualized on a log scale. Wilcox-Mann-Whitney tests were completed to test significant differences in target concentrations between clinical population distributions. *  p ≤ 0.05, ** p ≤ 0.01, *** p ≤ 0.001, **** p ≤ 0.0001.

**Fig 3:**
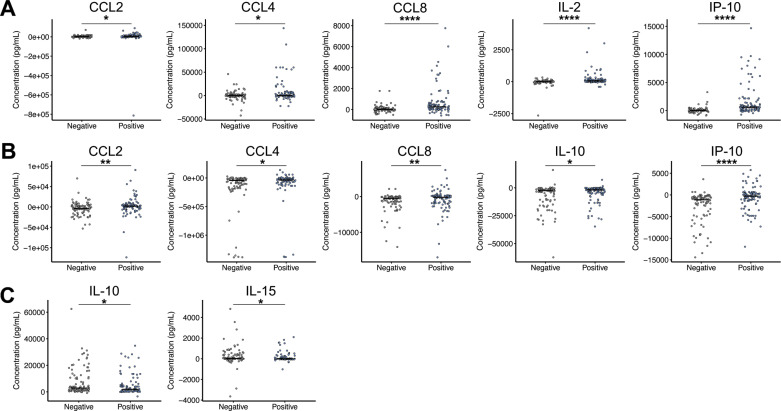
Significantly different normalized distributions between LTBI positive and negative patients. Normalized conditions of TB1-NIL (A), TB1-MIT (B), and MIT-NIL (C) were significantly different between clinical populations of LTBI+ and LTBI− patients in some measured targets. Median value of clinical population represented as a black line. Wilcox-Mann-Whitney tests were completed to test for significant differences in normalized target concentrations between clinical population distributions. *  p ≤ 0.05, ** p ≤ 0.01, *** p ≤ 0.001, **** p ≤ 0.0001.

The comparison of cytokine distributions between LTBI+ and LTBI− subjects in our population corroborates these selected important variables. All variables used to construct the reduced absolute cytokine-derived model have statistically different cytokine distributions between LTBI+ and LTBI− populations (Fig 2, S4 Table in S2 File). The greatest significance falls in the TB1 stimulated plasma, with IP-10 and CCL8 biomarkers being the most significantly different. Biomarkers IL-10 and IL-15 are significantly different across all three stimulation conditions. Similarly, the top five variables in the normalized cytokine-derived model have statistically different cytokine distributions between positive and negative populations (Fig 3, S4 Table in S2 File). The greatest significance falls in the TB1-related normalized cytokine values, with IP-10, CCL8, and IL-2 showing the greatest significance under TB1-NIL normalized conditions. IP-10 has the most distinction in population distributions between LTBI+ and LTBI− for two normalized conditions, TB1-NIL and TB1−MIT. Biomarkers IL-10 and IL-15 are only significantly different in the MIT-NIL normalized condition and are also the only biomarkers to have significant differences under this normalized condition.

### High risk stratification among LTBI+ subjects

We next aimed to differentiate patients classified as high risk of disease reactivation from all others in the LTBI+ population, which included patients at low risk of reactivation and those at an indeterminant risk. The LTBI+ subset of our study cohort constitutes the clinically relevant population for assessing the risk of reactivation. We used the clinical indicators described in the methods section to classify a patient as high TB reactivation risk given the information provided at sample collection and completed analysis with both absolute and normalized conditions for risk status, as was described for our LTBI status classification workflow.

**Fig 4:**
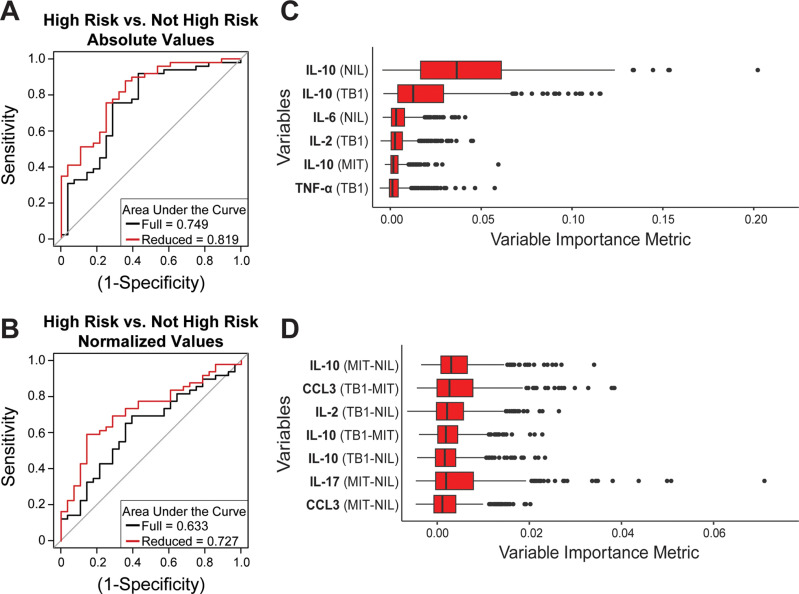
High-risk classification of LTBI positive patients using absolute and normalized stimulated cytokine concentrations. Random forest classification is represented as receiver operator characteristic (ROC) curves when using absolute (A) and normalized (B) stimulated biomarker concentrations. The most important variables from the full data set were identified through the variable importance metrics of absolute (C) and normalized (D) biomarker concentrations. Reduced data models were generated using the top biomarkers presented here. The variable importance metrics for all input variables in both models are presented in the Supplemental Information.

The absolute cytokine values from the stimulated plasma samples resulted in an accuracy of 74.9% (Fig 4A), and the normalized cytokine values resulted in an accuracy of 63.3% (Fig 4B) using the full data sets of 39 absolute or normalized variables, respectively. The variable importance metrics for the full model are provided for the absolute and normalized biomarkers in S5 and S6 Tables in S2 File, respectively, and the top variables were used to generate the reduced model. The refined absolute value-derived model used six variables (Fig 4C) and achieved an accuracy of 81.9%. With the normalized value input, the refined model resulted in an accuracy of 72.7% using seven variables (Fig 4D). Therefore, the refined absolute value-derived model had an ability to distinguish high-risk classified individuals from all others in the study with 9.2% greater accuracy than the normalized value-derived model.

Within the absolute cytokine value-derived model, the six variables used for the highest-performing reduced model consisted of just four protein biomarkers: IL-10, IL-6, IL-2, and TNF-α. All three stimulation conditions were present, with three emanating from TB1, two from NIL, and one from MIT. Concentrations of IL-10 after stimulation by all three conditions represent half of the top six variables, indicating a strong reliance on IL-10 for high-risk prediction. The variable importance metrics for the normalized value-derived model reveal a similar reliance on IL-10 and IL-2 associated variables, but overall, they have lower importance metrics. Other protein biomarkers, CCL3 and IL-17, are included in the reduced model using normalized conditions. Interestingly, across both models the top IL-10 biomarker is associated with non-Mtb-specific stimulation results, meaning the high-risk classification may be dependent on overall immune function of the LTBI+ patients.

### Low risk stratification among LTBI+ subjects

**Fig 5:**
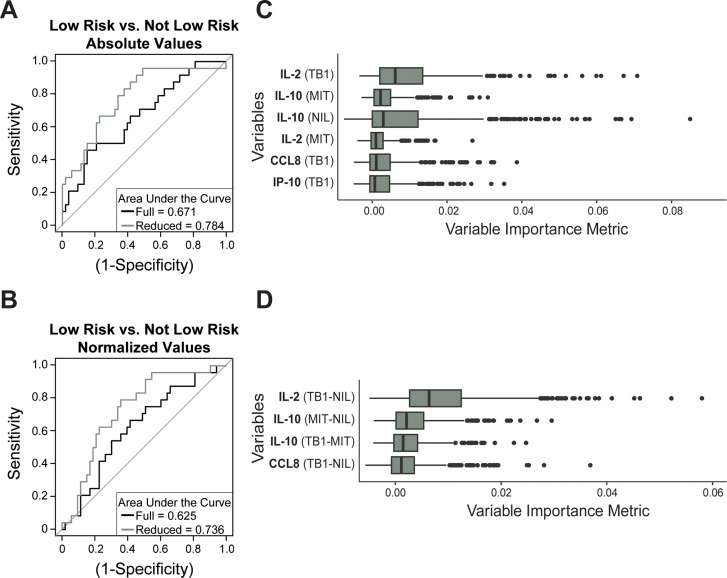
Low-risk classification of LTBI positive patients using absolute and normalized stimulated cytokine concentrations. Random forest classification is represented as receiver operator characteristic (ROC) curves when using absolute (A) and normalized (B) stimulated biomarker concentrations. The most important variables identified through the variable importance metrics using absolute (C) and normalized (D) biomarker concentrations were used to generate the reduced data models. The variable importance metrics for all input variables in both models are presented in the supplemental information.

We also aimed to differentiate patients classified as having a low risk of disease reactivation from all others in the LTBI+ population, which included patients at a high risk of reactivation and those at an indeterminant risk. We used the same clinical indicators and machine learning method employed in the high-risk classification. The absolute cytokine values from the stimulated QFT plasma samples resulted in an accuracy of 67.1% (Fig 5A), and the normalized cytokine values resulted in an accuracy of 62.5% (Fig 5B) using the full data sets of 39 absolute or normalized variables, respectively. The variable importance metrics for the full model are provided for the absolute and normalized biomarkers in S7 and S8 Tables in S2 File, respectively, and the top variables were used to generate the reduced model. The refined model used six variables in the absolute value-derived model (Fig 5C) and achieved an accuracy of 78.4%. With the normalized value input, the refined model resulted in an accuracy of 73.6% using four variables (Fig 5D). The refined absolute value-derived model had an ability to distinguish low-risk classified individuals from all others in the study with 4.8% greater accuracy than the normalized value-derived model with six input variables.

The six absolute-related variables used to generate the reduced model consisted of four different biomarkers, IL-2, IL-10, CCL8, and IP-10 with all three stimulations represented. The three unique biomarkers used to generate the reduced model with normalized conditions were similar, IL-2, IL-10, and CCL8, with all three normalized conditions present. Similar to the high-risk classification, there is a high reliance on IL-10 and IL-2 associated variables to accomplish the low-risk classification.

### Cytokine distributions between high and low risk patients

**Fig 6:**
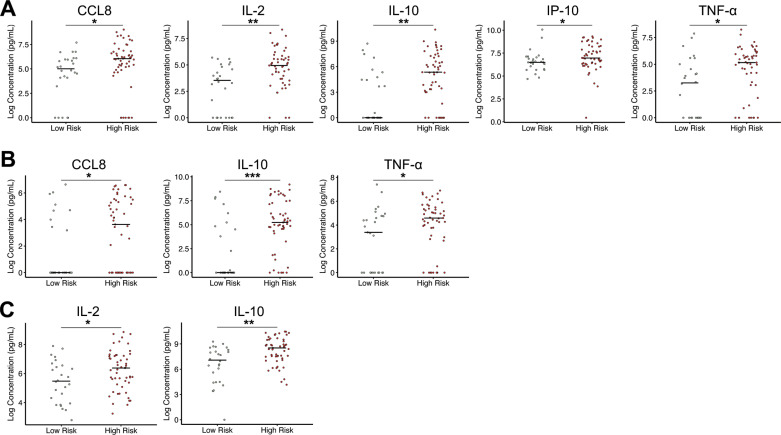
Significantly different absolute concentration distributions between high-risk and low-risk LTBI positive patients. Targets under stimulation conditions of TB1 (A), NIL (B), and MIT (C) that were significantly different between clinical populations of high and low risk LTBI+ patients are shown. Median value of clinical population represented as a black line. Data visualized on log scale. Wilcox-Mann-Whitney tests were completed to test for significant differences in normalized target concentrations between clinical population distributions. *  p ≤ 0.05, ** p ≤ 0.01, *** p ≤ 0.001, **** p ≤ 0.0001.

We next sought to further elucidate the differences of single biomarkers between the high and low reactivation risk populations. All LTBI+ patients who were classified as either high or low reactivation risk were included and WMW tests were completed to determine if there were significant differences in the distributions of the cytokines between high and low risk groups (S9 Table in S2 File). Targets with significant differences between absolute cytokine concentrations in the QFT stimulated plasma samples from high and low risk individuals are depicted in Fig 6. The ten variables with significantly different distributions between groups showed higher median concentrations in high-risk individuals than in low-risk individuals. IL-10 and IL-2 were imperative in classifying both bins using the random forest method, and both biomarkers also showed significance in absolute level discrimination. IL-10 distributions are significantly different across all three QFT stimulations, while IL-2 has significantly different distributions in TB1 and MIT stimulated plasma samples. Other variables that exhibit a significant difference between high and low risk groups include CCL8 (TB1 and NIL), TNF-α (TB1 and NIL), and IP-10 (TB1). WMW testing was also conducted using normalized variables. Only four significant differences resulted, with two associated with IL-10 and two with IL-2 (S9 Table in S2 File). This compliments the machine learning results, in that the absolute concentrations are better at distinguishing between risk groups than normalized concentrations.

## Discussion

This study successfully determined the diagnostic accuracy and identified biomarker signatures for LTBI and TB reactivation risk-based classifications by using QFT stimulated plasma samples coupled with microring resonator-based multiplexed cytokine profiling and machine learning feature selection methods. The 45-minute microring resonator multiplexed biomarker assay required just 210 μL of QFT plasma to simultaneously analyze thirteen cytokines at two sample dilutions. The multiplexed assay had been previously tested using cell stimulation supernatant samples, which are technically laborious to obtain [46,47]. Using QFT-stimulated plasma as the sample input places our method directly in the current TB diagnostic and clinical laboratory workflow, which decreases post-collection sample processing and lowers turnaround times. We successfully transitioned our assay workflow into plasma matrices and analyzed 495 QFT plasma samples.

The enrolled population for this study was representative of the type of individuals and patients commonly screened and evaluated for LTBI and TB in practices across the United States. This includes foreign-born individuals from high TB endemic areas, many with prior history of BCG vaccination, individuals with prior Mtb exposures, immunosuppressed patients, and healthcare workers with patient contact. A challenge to this type of study, and all LTBI studies, is that there is no gold standard diagnostic test for LTBI, and available diagnostic tests are imperfect, which introduces some inherent and unavoidable uncertainty into the initial clinical designations [83]. However, our study subjects were carefully selected to minimize heterogeneity in the study groups and current clinical diagnostic standards were rigorously followed [49]. Our study also included relevant clinical characteristics to analyze our immunoprofiling data, which has been suggested for optimal subject interpretation [84]. Simultaneous testing from the same blood samples and the use of internal and quality controls for all immunoassays also minimizes the risk of false-positive reactions and/or assay-related variability in our study results.

We report a multiplexed protein panel coupled with a reduced random forest model that differentiated LTBI+ and LTBI− patients with an accuracy of 90% or better using absolute or normalized biomarker values. The normalized cytokine values only improved the predictive accuracy marginally but displayed a greater separation in cytokine distributions between LTBI designations when compared to the absolute cytokine levels. The normalization could potentially account for patient-to-patient variability by subtracting the patient-specific cytokine concentrations induced by negative- and positive-control antigen stimulations. Using normalized conditions can improve the generalizability of the model and potentially reduce misclassification based on inherent variability in the basal immune response at the time of blood draw. Additionally, we combined both absolute and normalized cytokine concentrations into one model, resulting in 78 input variables per patient for classification (S3 Fig in S2 File). The accuracy did not improve from the models using absolute and normalized cytokine concentrations independently, suggesting that each individual data set is sufficient to reach the maximum accuracy given this set of biomarkers in our population. Although adding more variables potentially improves the amount of information contained in the data, the sample size in our study may not be able to support such a large model with high prediction accuracy. This phenomenon is commonly known as the curse of dimensionality in statistics.

The most important variables for making the LTBI status classification were similar between the absolute cytokine concentration-derived model and normalized cytokine level-derived model. The main classification biomarker across both methods was IP-10, a chemokine with high correlation to TB infection. Additionally, CCL8, IL-2, and IL-10 are present in the reduced models for both variable inputs and IL-15 is present in the absolute variable-derived model. These targets have been previously identified using other multiplexed biomarker analysis as having a relationship to LTBI or TB designation [36,37,46,47,68,71,85–92]. Our identification of diagnostic biomarkers with literature precedent lends credibility to our overall analytic and informatics approach. Notably, though, our results were achieved using a more rapid and automated assay format relative to previous analyses. Furthermore, all three stimulation conditions were represented in both absolute and normalized important variables, indicating that reliance on Mtb-specific stimulation responses and responses derived from inherent immune system activation are both important to classify LTBI patients. The control antigen stimulations are not typically used in LTBI classifications, but they provide added significance in our biomarker profiling results. Interestingly, absolute concentrations of IP-10, CCL8, and IL-2 are significantly higher in the LTBI+ population, but all remaining significantly different biomarkers are lower in this population. This indicates that increased levels of our top predictive variables *and* decreased levels of other predictive variables are being taken together by the model to perform the LTBI status classification.

Overall, both absolute and normalized models achieved 90% or better accuracy for differentiating LTBI+ patients from the remainder of our sample cohort. The normalized biomarker-derived model resulted in a marginally higher AUC and required fewer variables to achieve this classification. The normalized cytokine concentrations reveal great importance for IP-10, CCL8, IL-2, and IL-10 to identify LTBI+ subjects and we demonstrated the applicability of our multiplexed microring platform to rapidly analyze the residual QFT samples.

Protein-based risk classification is an important aspect of our study. Most TB reactivation risk classifications are based on RNA signatures. RNA is biochemically less stable than proteins and requires more specialized (RNAse-free) sample processing. Here, we integrated a risk of TB reactivation assessment into the same workflow as standard QFT testing and our LTBI classification method. The TB reactivation risk classification was completed within the LTBI+ population (n = 78) using the same biomarker measurements used for the LTBI status assessment model to classify LTBI+ patients at high or low risk of reactivation upon initial QFT assessment.

Compared to the LTBI classification, the risk classification models have less predictive accuracy. This could be due to a greater variability in subjects classified clinically as having a certain TB risk level. The classification of risk has no gold standard and relies on the analysis of many epidemiological and clinical factors determined with clinical expertise. However, the estimated annual and cumulative TB risk score based on unbiased multifactorial predicting modeling also confirmed the statistical differences in risk of TB reactivation between the low-risk and high-risk study groups (Table 1). Conversely, while LTBI status also does not have a defined gold standard, it is more objectively defined using clinical and quantitative metrics, including QFT results and additional testing. The lower predictive accuracy for risk classification could also be due to lower signal size, as the risk of reactivation assessment was restricted to the LTBI+ population. This population is likely more homogenous than the overall study population, and therefore less amenable to subcategorization via machine learning models.

Our interest in identifying orthogonal risk signatures for both low and high reactivation risk is based on an emerging role of how diagnostics could guide patient interventions and management. Although present guidelines recommend antibiotic therapy for LTBI+ subjects in the United States, concerns for targeted interventions and antibiotic stewardship motivate a need to identify subsets of patients that would benefit most from therapeutic intervention or longitudinal monitoring. Only 5 to 10% of LTBI+ patients reactivate to active TB, and the incidence of reactivation declines over time since the primary infection or exposure [4]. A biomarker-based signature that could establish a patient as being at high TB reactivation risk would provide a clear identification of patients most likely to benefit from preventive therapy. Conversely, a signature establishing low risk of TB reactivation at initial QFT sampling might allow those patients to be monitored for signs of potential emergence of disease, rather than the immediate initiation of therapy. This would reduce the chance of unnecessary adverse effects associated with antimicrobials in the large proportion of LTBI+ patients who will never progress to active TB. Furthermore, a diagnostic signature that develops over time could provide even more guidance for longitudinal monitoring of populations at a higher risk of contracting LTBI and progressing to active disease, such as LTBI+ patients, close contacts, or healthcare workers in TB endemic areas.

The high- and low-risk classifications in our study are inversely related but unique in terms of the goal population of the respective classification. The high-risk classification aims to identify the biomarker signature that uniquely identifies the high TB reactivation risk population from all others (low risk and indeterminant) in the LTBI+ cohort. Conversely, the low-risk classification aims to identify biomarkers that can specifically select the low-risk population from all others. Initially, we presumed that different variables or biomarkers would classify the different populations. The variable importance results revealed that similar variables are important for both classifications. The high overlap in important variables and statistically significant differences in concentration distributions between high and low reactivation risk groups implies the reactivation risk can be thought of as a spectrum, with concentrations of specific biomarker conditions responsible for differentiating both high and low risk individuals, as opposed to different protein biomarkers delineating the separate risk populations.

Both reactivation risk models had the greatest predictive accuracy when derived using the absolute cytokine values compared to normalized values. The global response of certain biomarkers, mainly IL-10 and IL-2, in the QFT stimulations is indicative of the immune surveillance to infection. This host response led to greater designation accuracy and greater significant differences between cytokine levels in clinical populations, in comparison to the normalized conditions. Absolute values of IL-10 and IL-2 account for four of the six variables in the reduced models for both classifications. All the IL-10 and IL-2 associated important variables show statistically significant differences in cytokine concentration distributions between respective clinical populations, with higher concentrations in the population at high risk of TB reactivation. The higher levels of IL-10 in the group at a high risk of TB reactivation may be indicative of an attenuation of the Th1 response that inhibits the formation of mature fibrotic granuloma through suppression of macrophage and dendritic cell functions, leading to a disruption in the dynamic structure of the granuloma [93]. The higher levels of IL-2 may be associated with antigenic memory response and the selected Th2 induced IL-10 response [23]. The increased levels of IL-10 and IL-2 in our LTBI+ group at a high reactivation risk suggests they could be mediators of TB reactivation and disease progression, and our results indicate they can differentiate our LTBI+ subject population by clinically designated reactivation risk at the time of QFT sample collection.

The normalized variables led to a marginally better predictive accuracy for LTBI status designation, while the absolute value variables proved to be best for the risk prediction model. In comparing the protein biomarkers necessary to construct the best reduced models across all three designations, a group of just six proteins stands out, namely IP-10, CCL8, IL-2, IL-10, IL-6, and TNF-α. This biomarker reduction has implications for future work on reducing panel size towards developing smaller biomarker panels amenable across multiple cytokine detection platforms. However, it is important to note that our more highly multiplexed technology was essential in allowing us to identify this reduced number of target analytes. All three QFT stimulations were in each of the reduced variable lists, so eliminating a stimulation is not supported by our results.

IFN-γ is a cytokine heavily utilized in TB diagnostics. However, IFN-γ is not a selected biomarker in any of our reduced models. IFN-γ is a strong predictor of active TB, as IFN-γ is one of the drivers of inducing or clearing granuloma formation [7,26]. However, recent literature details specific instances where LTBI equilibrium is IFN-γ independent with all other immunological elements within comparable ranges. There are multiple factors that could account for this established TB biomarker not contributing to our LTBI clinical significance. One explanation is that the dynamic equilibrium that is yielding the latent Mtb infection state and the propensity for it to shift towards TB progression either does not rely on IFN-γ production, or that other peripheral immune pathways contribute more to this dynamic shift. Alternatively, it could be attributed to the clinical designations themselves, where the LTBI+ designation is determined by the QFT IFN-γ results; therefore, heterogeneous populations in both clinical categories lead to an insignificant contribution.

A limitation of our efforts to selectively identify LTBI+ populations is the near absence of active TB patients within the study population. Developing diagnostics that can distinguish LTBI+ from active TB populations is important, as these groups traditionally exhibit similar IGRA and molecular testing results. However, methods to detect active TB infection are well established and the low frequency of active TB in our study cohort accurately reflects the overall population of individuals receiving QFT testing at the Mayo Clinic. This implies there is a greater need in this population to distinguish LTBI+ individuals from other QFT tested patients than to distinguish LTBI+ and active TB individuals. Nevertheless, future work will aim to include an active TB population, including early forms of TB, to test the sensitivity and specificity of classifying LTBI+ patients from active TB patients. Another limitation of our approach is that while the LTBI status is more objectively defined, the risk classifications involve more subjective assessments. This can impart high variability in patient classification and lead to suboptimal predictive accuracies. However, these limitations represent a significant challenge across all LTBI diagnostic research, and the absence of gold standards highlights the urgency for continued research efforts. Finally, while cytokine biomarker panels containing a greater number of targets are commercially available, many still require manual manipulation which can impart operator variability. The biosensor platform employed in this study was limited to sixteen analytes, including control markers. However, each analyte was measured in four technical replicates and all samples were processed through automated microfluidic handling.

A goal of our study was to use QFT samples collected at an initial screen to classify risk of reactivation from LTBI to active TB. Patients with high risk have a greater potential to progress to active TB, while those with low risk have a lower potential to reactivate. Treatment and monitoring for the two populations can look different. Prognostic biomarkers of reactivation or disease progression have been identified through longitudinal studies that monitor biomarker profiles in patients before and after they progress to disease. These biomarkers are useful but reactivating to active TB over the course of a study can be related to environmental or host factors not captured with the biomarker profiles. Additionally, if treatments are provided to those identified as LTBI+ early in the study, the prognostic biomarkers are limited to a population of treated patients, rather than to all LTBI+ individuals. Furthermore, most work is focused on progression biomarkers and does not aim to find non-progression biomarkers. Our work was not focused on using progression to active TB as an endpoint, rather, we aimed to use biomarkers to classify patients by reactivation risk upon initial QFT sample collection. The implications of identifying these biomarkers are that they can be generalized to all LTBI+ patients through larger, multi-center studies and could be used to adjust patient monitoring schedules. Those identified as low risk could receive specific management recommendations with less frequent monitoring, which would be more feasible for individuals, decrease the risk of medication side effects, and potentially save time and resources. Future work in this ongoing study includes increasing patient cohort size, inclusion of subjects with active TB, those with early forms of TB, and recent close contact of pulmonary TB patients, and working with populations in high-TB burden environments to apply our workflow in high need areas.

## Conclusion

This work successfully applied a microring resonator multiplexed sensing platform within a clinically relevant workflow to the TB diagnostic space. Our goals were to use the concentrations of thirteen cytokines in QFT stimulated plasma to identify biomarker signatures that could select LTBI+ subjects and classify their risk of reactivation to active TB. Within one 13-plex cytokine biomarker panel, we demonstrated accuracies over 90% to differentiate LTBI+ patients and accuracies of nearly 80% for stratifying those patients as high or low risk of reactivation. Variable importance metrics highlighted a significant reliance on various normalized conditions of IP-10, CCL8, IL-10, and IL-2 for making the LTBI classification, while concentrations from various absolute concentrations of IL-10 and IL-2 were extremely important for classifying LTBI + patients as high or low TB reactivation risk. Our work employed a rapid technique with high multiplexing capabilities to profile clinically relevant samples and identify biomarkers of importance consistent with previous literature. The inclusion of low TB reactivation risk designation in conjunction with high TB reactivation risk designation provides a unique way to stratify LTBI+ patients with potential future implications in clinical monitoring and treatment management.

## Supporting information

S1 File Supporting information file. The supporting information file contains additional experimental details, including a multiplexed calibration curve for all thirteen targets in the panel; example data trace from a patient specimen; LTBI classification ROC curves; antibody and reagent information; variable importance metrics (VIMs) for absolute and normalized cytokine values, median values of absolute and normalized cytokine levels for different clinical designations.
(CSV)

S2 File Supporting information file. The supporting information file contains additional experimental details, including a multiplexed calibration curve for all thirteen targets in the panel; example data trace from a patient specimen; LTBI classification ROC curves; antibody and reagent information; variable importance metrics (VIMs) for absolute a normalized cytokine values. The description for the CSV file should be "Clinical subject data and median values of absolute and normalized cytokine levels for different clinical designations.
(DOCX)
