# Supplementary material for: Multiplexed cytokine profiling identifies diagnostic signatures for latent tuberculosis and reactivation risk stratification
Source: PLoS One. 2025 Apr 9;20(4):e0316648. doi: 10.1371/journal.pone.0316648 (PMC11981658; doi:10.1371/journal.pone.0316648)
Supplement: S2 File — The supporting information file contains additional experimental details, including a multiplexed calibration curve for all thirteen targets in the panel; example data trace from a patient specimen; LTBI classification ROC curves; antibody and reagent information; variable importance metrics (VIMs) for absolute a normalized cytokine values. The description for the CSV file should be "Clinical subject data and median values of absolute and normalized cytokine levels for different clinical designations. (DOCX) [file pone.0316648.s002.docx]

## Multiplexed cytokine profiling identifies diagnostic signatures for latent tuberculosis and reactivation risk stratification

Krista Meserve^1^, Cole A. Chapman^1^, Mingrui Xu^2^, Haowen Zhou^3^, Heather M. Robison^1^, Heather R. Hilgart^4^, Pedro P. Arias-Sanchez^5^, Balaji Pathakumari^5^, Manik R. Reddy^1^, Kale A. Daniel^5^, Thomas M. Cox^5^, Courtney L. Erskine^6^, Paige K. Marty^5^, Mounika Vadiyala^5^, Snigdha Karnakoti^5^, Virginia Van Keulen^5,6^, Elitza Theel^4^, Tobias Peikert^5^, Colleen Bushell^7^, Michael Welge^7^, Rafael Laniado-Laborin^8¶^, Ruoqing Zhu^2¶^, Patricio Escalante^5¶^, Ryan C. Bailey^1¶*^

^1^ Department of Chemistry, University of Michigan, Ann Arbor, Michigan, United States of America

^2^ Department of Statistics, University of Illinois Urbana-Champaign, Champaign, Illinois, United States of America

^3^ Department of Statistics, University of Virginia, Charlottesville, Virginia, United States of America

^4^ Department of Laboratory Medicine, Mayo Clinic, Rochester, Minnesota, United States of America

^5^ Department of Medicine, Division of Pulmonary and Critical Care Medicine, Mayo Clinic, Rochester, Minnesota, United States of America

^6^ Department of Immunology, Mayo Clinic, Rochester, Minnesota, United States of America

^7^ National Center for Supercomputing Applications, University of Illinois Urbana-Champaign, Urbana, Illinois, United States of America

^8^ Clinica y Laboratorio de Tuberculosis, Facultad de Medicina y Psicologia, Hospital General Tijuana, Universidad Autonoma de Baja California, ISESALUD, Tijuana, Baja California, Mexico

^*^ Corresponding author

E-mail: [ryancb@umich.edu](mailto:ryancb@umich.edu) (RCB)

^¶^ These authors contributed equally to this work.

**Microring Resonator Sensor Methodology**

### *Silicon photonic microring resonator technology and instrumentation*

Silicon photonic microring resonators are a type of whispering-gallery mode sensor in which light continually circulates within a fabricated microstructure, resulting in an evanescent field extending above the microring surface.[1] Light couples into the microring waveguide at a wavelength sensitive to the effective refractive index (RI) sampled by the waveguide evanescent field, according to the following equation:

$$\lambda= \frac{2\pi r}{m}n_{eff}$$

where *λ* is the wavelength of light, *m* is an integer, *r* is the radius of the microring waveguide, and $n_{eff}$ is the effective refractive index of the optical mode.[2,3] Capture probes (e.g., antibodies) covalently attached to the microring resonator bind to and localize specific target biomolecules to the sensor surface, which alters the effective refractive index within the sensing region. The altered refractive index induces a change in the resonant wavelength, which is measured during a binding assay. Using calibration curves, the overall wavelength shift over the course of an assay can be correlated to the concentration of each target biomolecule in a sample.[4,5] Binding assays utilizing the microring structure exhibit a low coefficient of variation, and multiple microring sensors may be readily fabricated on a single sensing chip for multiplexed analyte quantitation.

The presented cytokine biomarker data was collected using the Genalyte Matchbox instrument (Genalyte, Inc., San Diego, CA) and silicon sensor chips (Genalyte, Inc. San Diego, CA). The silicon sensor chips were 4 x 6 mm in size and fabricated with 128 individual microrings. Microrings were arranged in clusters of four, and sixteen clusters were distributed across each of two identical linear arrays. This sensor chip layout allows for the simultaneous analysis of up to sixteen different analytes in two samples, with four technical replicates of each analyte. The multiplexed sensor chips were housed in injection molded cartridges to create two sealed microfluidic channels along the top of the linear microring arrays. When inserted into the instrument, a fully automated microfluidic path is created that delivers the sample and all reagent liquids from a 96-well plate across the surface of the sensor chip at 30 μL/min before being directed to waste. The sensor chip and cartridge unit were discarded after each sample to eliminate potential carryover between samples.

### *Sensor chip preparation*

As previously described, capture antibodies for each biomarker were covalently linked to clusters of four microring sensors through silanization with (3-aminopropyl)triethoxysilane (APTES), followed by introduction of a homo-bifunctional linker, bis(sulfosuccinimidyl) suberate (BS3), and the antibody of interest using precision, piezoelectric spotting by Genalyte, Inc.[4–6] The sensor chips were blocked with bovine serum albumin (BSA) containing buffer, dry coated for antibody stability, and stored in a desiccator at 4°C. Prior to use, the chips were rinsed with deionized water to remove the dry coat and fitted into the injection molded cartridges.

### *Assay parameters*

A functionalized chip was inserted into the instrument and running buffer, 1X phosphate buffered saline with 0.5% BSA (PBS-BSA), was flowed across the chip to equilibrate the surface for five minutes. The data collection then began with running buffer flowing consistently for two minutes to collect baseline signal. The following assay reagents were then flowed across sequentially: sample of interest in plasma matrix (7 mins), buffer rinse (2 mins), mixture of biotinylated tracer antibodies (1-2 μg/mL, 7 mins), a buffer rinse (2 mins), streptavidin horse-radish peroxidase (SA-HRP, 4 μg/mL, 7 min), a buffer rinse (2 mins), 4-chloro-1-napthol (4CN, stock concentration, 7 mins), and a final buffer rinse (3 mins), for a total assay time of 39 minutes. The buffer rinses remove any unbound material before the next reagent is introduced. The final reagent, 4CN, reacts with the localized HRP to form an insoluble precipitate within the evanescent field, amplifying the resonant wavelength shift to allow lower detection limits and broader sensing regions. To quantify relative shifts in each sample, the signal immediately before the 4CN step (t=29 min) was subtracted from the signal at the end of the final buffer rinse (t=39 min) to obtain the net shift (Δpm) for each individual target.

### *Assay panel optimization and multiplexed calibrations*

Each biomarker was individually optimized to determine optimal tracer antibody concentration and appropriate standard concentration that allowed for construction of an eight-point, four-parametric calibration curve that ranges from a saturating signal to baseline signal. Prior to multiplexing the biomarkers, each pair of capture antibody and standard/tracer antibody was tested for cross-reactivity. Using a checkerboard method, each individual standard/tracer antibody pair was flowed across each capture antibody to ensure that capture antibodies produce a response only to their respective standard/tracer antibody pair. Additionally, optimized sandwich assays were tested in human plasma to ensure detection efficacy in the biologic matrix of interest. New reagent lots introduced during the study were tested before use in sample analysis.

Calibration of the 13-plex immunoassay was completed in a multiplexed format in two relevant background matrices (50% plasma and 10% plasma). Seven serial dilutions from a saturating analyte concentration and a matrix blank were analyzed in the assay format described above and yielded an eight-point calibration curve relating standard concentrations to relative resonance wavelength shift. Net resonance wavelengths (∆pm) were plotted as a function of standard concentration and fit to a four parametric logistic function, as described previously.[5] Limits of detection (LOD) and quantification (LOQ) were defined as the blank signal plus three times and ten times the standard deviation of the blank, respectively. New calibrations were constructed for each batch of sensor chips. At least three calibrations were completed per matrix and averaged over the usage lifetime of each chip batch to account for temporal variation in the assay signal. The averaged calibrations were used to construct a final four parametric calibration curve fit for each target that was subsequently applied to convert net resonance wavelength shifts to analyte concentrations in the patient samples.

## Machine Learning Methodology

### *Random forest algorithm*

With clinical data represented as $(X, Y),$where $X = (X_{1}, X_{2}, \cdot, X_{p})$ denotes the biomarker features and Y signifies the clinical designation (e.g., LTBI, high risk, or low risk), we employ the random forest algorithm to construct a classification model between the features and clinical designations.[7,8] The utilization of random forest offers several statistical advantages. First, it provides interpretable insights into the classification task, enabling practitioners to understand the factors driving the predictions. Second, random forest excels in handling high-dimensional data, allowing for the exploration of nonlinear effects and interactions among covariates. This capability is particularly valuable in scenarios where traditional linear models may be insufficient. Third, random forest incorporates a built-in mechanism for ranking the most important predictors, facilitating the identification of key variables influencing the prediction model.

The random forest algorithm, an ensemble classifier, operates by combining multiple tree-based estimators, $\{T_{i}\}$, through the bagging method for sample and variable selection. Each classification tree within the ensemble is constructed by drawing bootstrap samples from the training data, with variables sampled at each node split. The prediction output of the random forest is determined by aggregating the predictions from each individual tree, typically through averaging or majority voting. Compared to single tree-based estimators, random forests offer enhanced estimation accuracy due to the combination of bagging and aggregation, which effectively reduces variance and improves predictive performance. This advantage is particularly pronounced in high-dimensional datasets, where variable bagging helps to mitigate the impact of noise introduced by non-informative variables, thereby yielding more robust and reliable predictions.

### *Parameter tuning and measurement*

The tuning parameters of the random forest algorithm, namely the size of the random sample of exposures used at each split (mtry), the number of trees (ntree), and the minimum number of observations in the final nodes (nodesize), were optimized to enhance prediction performance. An exhaustive search was conducted to determine the optimal parameter values. To assess the prediction performance of the random forest algorithm, we employed the Receiver Operating Characteristic (ROC) curve and computed the Area Under the Curve (AUC). The ROC curve provides a graphical representation of the trade-off between true positive rate and false positive rate across different classification thresholds, while the AUC quantifies the model’s discriminative ability. Utilizing the out-of-bag (OOB) sample in each tree, we evaluated the performance of various parameter settings. Specifically, we selected the parameter configuration that yielded the highest performance in terms of AUC using the OOB sample. This approach ensures robust model selection and optimization, leading to improved predictive accuracy and generalization capability of the random forest algorithm.

### *Sampling strategy with repeated entries*

This clinical data contains an identification (ID) label for each data record. Multiple records collected for the same subject in our longitudinal study violates the independence assumption of common statistical models. Often mixed effect models[9,10] and other approaches [11,12] are utilized to address this issue. However, existing methods cannot be incorporated into the random forest splitting rule. This unique dataset serves two crucial purposes, measurement of classification accuracy and assessment of variable importance.

To remove the dependencies of samples in both the model fitting and variable importance evaluation, we propose a new sampling strategy. At the outset of random forest construction, observations are sampled from the original dataset for each tree. During this stage, unique records are sampled for all IDs as inputs for individual trees, while ensuring that no repeated records are used from the same ID. When evaluating variable importance, bootstrapping is applied to the unique dataset. It is ensured that the same identifier from bootstrapping remains in the same group, either in-bag or out-of-bag. This approach maintains consistency and integrity in the assessment of variable importance, which will be illustrated in the following supplemental section, facilitating robust analysis and model refinement.

### *Variable selection with variable importance*

Random forests offer a robust framework for variable selection and dimension reduction through the assessment of variable importance. Variables with higher importance rankings are considered more influential in prediction tasks. In random forests, variable importance is quantified as the difference in prediction error when a variable is perturbed compared to when it remains unchanged.[8,13,14] This assessment is conducted within each individual tree using out-of-bag data, which is not sampled during training. To measure variable importance, prediction accuracy is estimated by fitting the out-of-bag samples into the trained tree-based model. Subsequently, the values of the variable in the out-of-bag data are shuffled while keeping other variables constant. The variable importance measure is then calculated as the decrease in prediction accuracy of the shuffled out-of-bag data compared to the original data. In cases where a variable demonstrates minimal predictive power, shuffling may lead to a marginal increase in accuracy due to random noise, resulting in trivial negative importance scores that can be effectively considered as zero importance.

Our primary objective at the outset is to identify informative variables. To achieve this, we initiate the first stage by training the random forest using the complete set of biomarker features. It is critical to ensure that data IDs are uniquely sampled from all records within the dataset, preserving the integrity of the analysis. Leveraging the utilization of out-of-bag samples, we extract variable importance scores from each tree in the ensemble. During the calculation of these scores, we maintain consistency by ensuring that the same IDs from bootstrapping are consistently allocated to either the in-bag or out-of- bag samples. These scores are subsequently treated as bootstrapped samples, facilitating the construction of empirical confidence intervals for assessing the variable importance of each biomarker feature. This meticulous approach ensures robustness in the evaluation and selection of influential variables for the analysis. Transitioning to the second stage, our focus shifts towards identifying significant variables. Utilizing a specified confidence level, we conduct one-sided testing to determine whether the variable importance is positive, utilizing the confidence intervals established in the first stage. Subsequently, employing the selected variables along with the complete set of IDs, we construct a new random forest model, thereby refining our predictive framework.

| **Algorithm 1:** Random Forest for Clinical Designation Classification |
| --- |
| **Input:** Clinical data (X, Y), Threshold Level α  **Output:** Random Forest $\mathcal{T}$, Selected Variables ${\{X_{i}\}}_{i\in I}$ |
| 1. Sample unique records for all IDs from $(X, Y)$ to create a unique dataset $(X_{U}, Y_{U});$ |
| 1. Train a random forest with the unique dataset $(X_{U}, Y_{U})$ and tune the parameters (mtry, nodesize, ntree) based on out-of-bag samples; |
| 1. With tuned parameter, calculate the variable importance score $s_{k}^{i}$ for each variable $X_{i}$ from each tree $T_{k}$, $k$ = 1, 2, · · ·, *ntree* and enforce the same ID from bootstrapping to be in-bag or out-of-bag; |
| 1. Build empirical confidence interval $S_{i}$from ${{\{s}_{k}^{i}\}}_{k=1, 2,\cdot\cdot\cdot ntree}$ for feature $X_{i}$; |
| 1. Select variables $X_{i}$ when (1 − α) portion of the variable importance interval $S_{i}$ exceeds 0; |
| 1. Repeat steps 1 and 2 using the selected variables ${\{X_{i}\}}_{i\in I}$ to obtain the final random forest $\mathcal{T}$. |

## Supplemental Figures


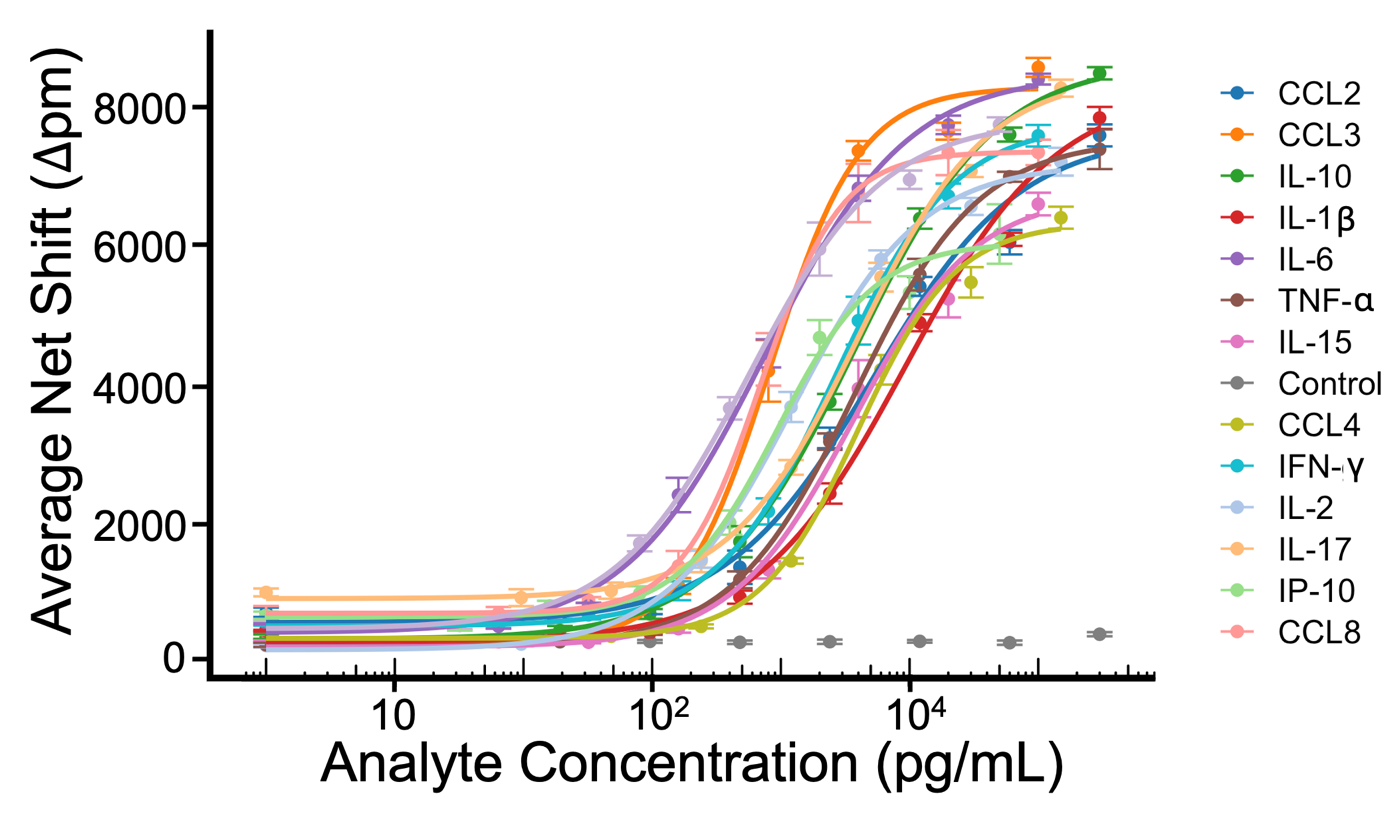


###

### Supplemental Figure 1. Example of a multiplexed calibration curve for all thirteen targets in the panel in a 50% plasma matrix. The eight-point calibration curves were done in triplicate for each batch of spotted chips. The triplicate calibrations were completed over the course of running samples to encompass any temporal variation in the assay. Data is shown as an average and standard deviation of the three calibrations. Calibration curve parameters were extracted from a four parametric fit.


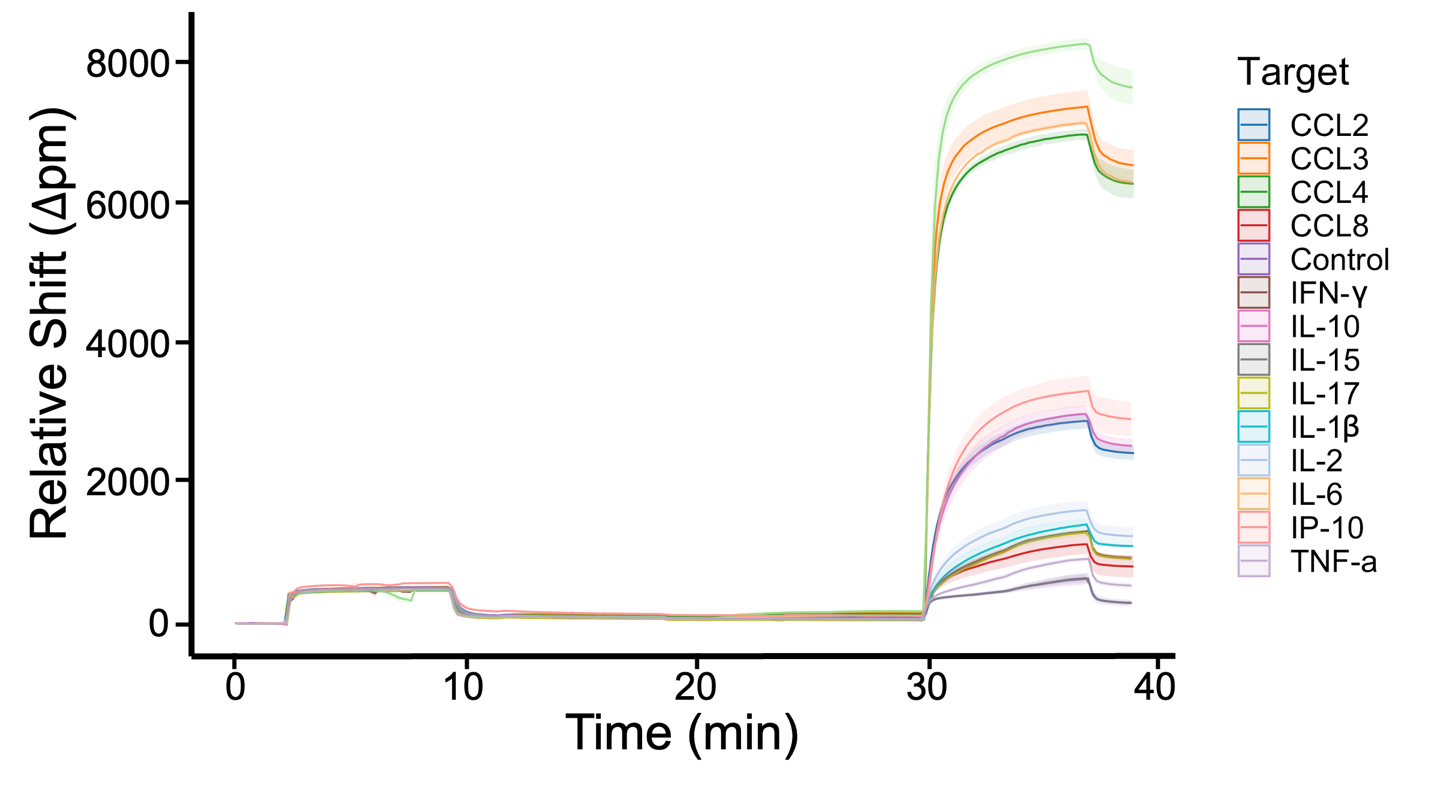


Supplemental Figure 2. Example of a data trace from a patient plasma sample. The real-time trace output from the instrument facilitates experiment monitoring at each step of the immunoassay. Immunoassay steps were as follows: buffer rinse (2 mins) sample of interest (7 mins), buffer rinse (2 mins), mixture of biotinylated tracer antibodies (1-2 μg/mL, 7 mins), buffer rinse (2 mins), streptavidin horse-radish peroxidase (SA-HRP, 4 μg/mL, 7 min), buffer rinse (2 mins), 4-chloro-1-napthol (4CN, stock concentration, 7 mins), and a final buffer rinse (3 mins). The net shifts utilized to quantitate each target were calculated as the relative shift at 39 minutes minus the relative shift at 29 minutes. Traces and shaded area around each trace represent the average and standard deviation of the four technical ring replicates per target, respectively.


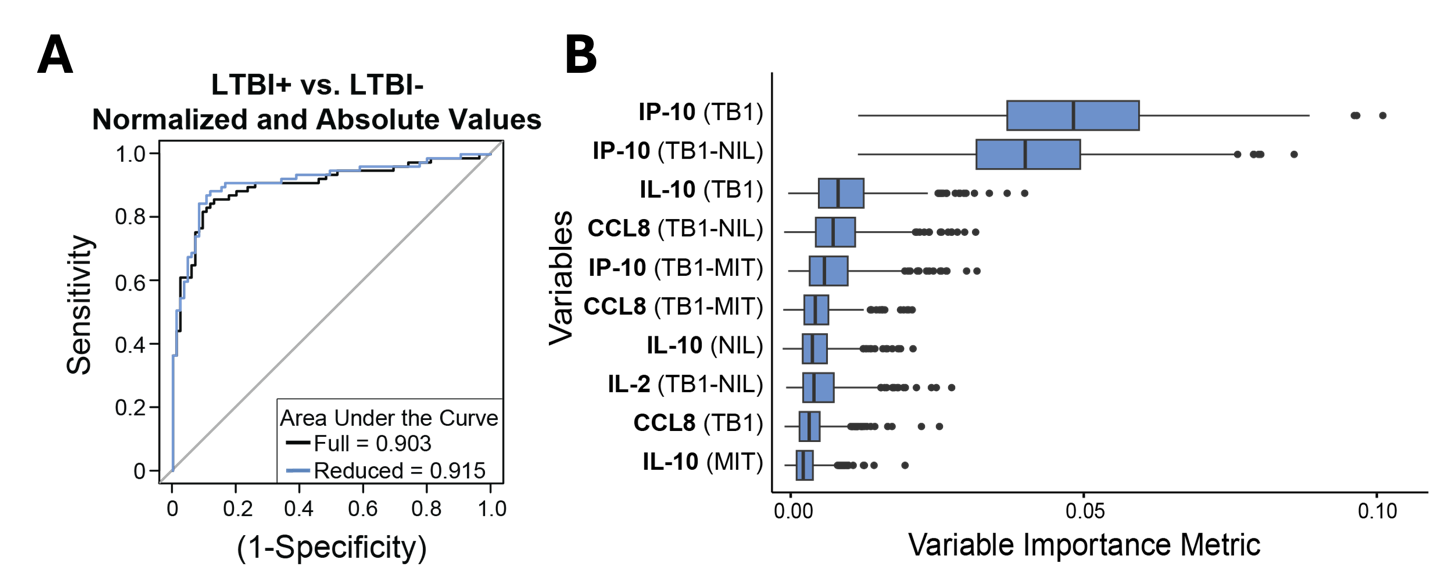


Supplemental Figure 3. LTBI classification using both absolute and normalized stimulated cytokine concentrations. ROC curve (A) results from the model developed to distinguish LTBI positive and LTBI negative patients using combined absolute and normalized conditions. The top ten most important variables identified through variable importance metrics are presented (B).

## Supplemental Tables

### Supplemental Table 1. Antibody and standard reagent information.

| **Target** | **Assay Context** | **Clonality and Clone** | **Reactivity** | **Host/Isotype** | **Supplier** | **Catalog number** | **Running Concentration** |
| --- | --- | --- | --- | --- | --- | --- | --- |
| **CCL2** | Capture Antibody | Monoclonal, 5D3-F7 | Human | Mouse/IgG1 | ThermoFisher Scientific | 14-7099 | 0.25 mg/mL |
|  | Human Recombinant Protein |  |  |  | ThermoFisher Scientific | RP-8648 | 300 ng/mL |
|  | Biotinylated Detection Antibody | Monoclonal, 2H5 | Human, Mouse, Rat | Armenian hamster/IgG | ThermoFisher Scientific | 13-7096 | 2 μg/mL |
| **CCL3** | Capture Antibody | Monoclonal, 14215 | Human | Mouse/IgG_2A_ | R&D Systems | MAB670 | 0.25 mg/mL |
|  | Human Recombinant Protein |  |  |  | R&D Systems | 270-LD | 100 ng/mL |
|  | Detection Antibody (biotinylated in-house) | Monoclonal, 93321 | Human | Mouse/IgG | R&D Systems | MAB270 | 1 μg/mL |
| **CCL4** | Capture Antibody | Monoclonal, 24006 | Human | Mouse/IgG_2B_ | R&D Systems | MAB271 | 0.25 mg/mL |
|  | Human Recombinant Protein |  |  |  | R&D Systems | 270-LD | 150 ng/mL |
|  | Detection Antibody (biotinylated in-house) | Monoclonal, 24014 | Human | Mouse | R&D Systems | CUST01702 | 2 μg/mL |
| **CCL8** | Capture Antibody | Monoclonal, 35509 | Human | Mouse/IgG | R&D Systems | MAB281 | 0.25 mg/mL |
|  | Human Recombinant Protein |  |  |  | R&D Systems | 281-CP | 100 ng/mL |
|  | Detection Antibody (biotinylated in-house) | Polyclonal | Human | Goat IgG | R&D Systems | BAF281 | 2 μg/mL |
| **IFN-γ** | Capture Antibody | Monoclonal, 1-D1K | Human | Mouse/IgG_1_ | Mabtech | 3420-3 | 0.25 mg/mL |
|  | Human Recombinant Protein |  |  |  | ThermoFisher Scientific | RIFNGSO | 100 ng/mL |
|  | Biotinylated Detection Antibody | Monoclonal, 7-B6-1 | Human, Non-human primates | Mouse/IgG_1_ | Mabtech | 3420-6 | 1 μg/mL |
| **IL-1β** | Capture Antibody | Monoclonal, CRM56 | Human | Mouse/IgG_1_ | ThermoFisher Scientific | 14-7018 | 0.25 mg/mL |
|  | Human Recombinant Protein |  |  |  | ThermoFisher Scientific | RIL1BI | 300 ng/mL |
|  | Biotinylated Detection Antibody | Monoclonal, CRM57 | Human | Mouse/IgG_2B_ | ThermoFisher Scientific | 13-7016 | 2 μg/mL |
| **IL-2** | Capture Antibody | Monoclonal, 5344 | Human | Mouse/ IgG_1_ | BD Bioscience | 555051 | 0.25 mg/mL |
|  | Human Recombinant Protein |  |  |  | BD Bioscience | 554603 | 150 ng/mL |
|  | Biotinylated Detection Antibody | Monoclonal, B33-2 | Human | Mouse/ IgG_1_ | BD Bioscience | 555040 | 2 μg/mL |
| **IL-6** | Capture Antibody | Monoclonal, MQ2-13A5 | Human | Rat/ IgG_1_ | ThermoFisher Scientific | 16-7069 | 0.25 mg/mL |
|  | Human Recombinant Protein |  |  |  | ThermoFisher Scientific | RP8619 | 100 ng/mL |
|  | Biotinylated Detection Antibody | Monoclonal, MQ2-39C3 | Human | Rat/IgG_2A_ | ThermoFisher Scientific | 13-7068 | 2 μg/mL |
| **IL-10** | Capture Antibody | Monoclonal, JES3-9D7 | Human | Rat/ IgG_1_ | ThermoFisher Scientific | 16-7108 | 0.25 mg/mL |
|  | Human Recombinant Protein |  |  |  | ThermoFisher Scientific | PHC0105 | 300 ng/mL |
|  | Biotinylated Detection Antibody | Monoclonal, JES3-12G8 | Human | Rat/IgG_2A_ | ThermoFisher Scientific | 13-7109 | 2 μg/mL |
| **IL-15** | Capture Antibody | Monoclonal, BH1509 | Human | Mouse/ IgG_1_ | Biolegand | 515001 | 0.25 mg/mL |
|  | Human Recombinant Protein |  |  |  | R&D Systems | 247ILB | 100 ng/mL |
|  | Biotinylated Detection Antibody | Monoclonal, BH1543 | Human | Mouse/IgG_1_ | Biolegand | 515104 | 2 μg/mL |
| **IL-17** | Capture Antibody | Monoclonal, MT44.6 | Human | Mouse/IgG_1_ | Mabtech | 3520-3 | 0.25 mg/mL |
|  | Human Recombinant Protein |  |  |  | Biolegand | 570502 | 150 ng/mL |
|  | Biotinylated Detection Antibody | Monoclonal, MT504 | Human, Non-human primate, cow, sheep, horse, pig, dog | Mouse/IgG_1_ | Mabtech | 3520-6 | 2 μg/mL |
| **IP-10** | Capture Antibody | Monoclonal, 4D5/A7/C5 | Human | Mouse/IgG_2A_ | BD Bioscience | 555046 | 0.25 mg/mL |
|  | Human Recombinant Protein |  |  |  | BD Bioscience | 551130 | 50 ng/mL |
|  | Biotinylated Detection Antibody | Polyclonal | Human | Goat/IgG | R&D Systems | BAF266 | 0.5 μg/mL |
| **TNF-α** | Capture Antibody | Monoclonal, MAb1 | Human | Mouse/IgG_1_ | Biolegend | 502801 | 0.25 mg/mL |
|  | Human Recombinant Protein |  |  |  | Biolegend | 570102 | 300 ng/mL |
|  | Biotinylated Detection Antibody | Monoclonal, MAb11 | Human | Mouse/IgG_1_ | Biolegend | 502904 | 2 μg/mL |

### Supplemental Table 2. Variable importance metrics (VIM) of the full model for LTBI classification with absolute values as the model input.

| Input Variable | Mean VIM | Median VIM |
| --- | --- | --- |
| IP-10 (TB1) | 1.14E-01 | 1.12E-01 |
| IL-10 (TB1) | 1.66E-02 | 1.40E-02 |
| IL-10 (NIL) | 9.41E-03 | 7.51E-03 |
| CCL8 (TB1) | 6.96E-03 | 4.75E-03 |
| IL-2 (TB1) | 5.93E-03 | 4.30E-03 |
| IL-15 (MIT) | 3.17E-03 | 2.53E-03 |
| IL-15 (NIL) | 2.93E-03 | 2.24E-03 |
| IL-15 (TB1) | 2.76E-03 | 2.06E-03 |
| IL-10 (MIT) | 3.48E-03 | 2.03E-03 |
| CCL8 (MIT) | 1.20E-03 | 8.30E-04 |
| CCL2 (MIT) | 8.51E-04 | 6.04E-04 |
| IL-1β (MIT) | 1.04E-03 | 5.79E-04 |
| CCL4 (MIT) | 5.74E-04 | 3.45E-04 |
| IL-17 (MIT) | 9.96E-04 | 2.35E-04 |
| TNF-α(TB1) | 1.05E-03 | 2.29E-04 |
| IL-17 (NIL) | 9.53E-04 | 1.52E-04 |
| TNF-α(MIT) | 2.57E-04 | 9.50E-05 |
| IL-1β (TB1) | 1.93E-04 | 3.35E-05 |
| IL-1β (NIL) | 4.12E-04 | 3.04E-05 |
| CCL2 (TB1) | 1.88E-04 | -6.86E-07 |
| CCL3 (MIT) | 2.77E-04 | -1.69E-06 |
| CCL3 (NIL) | 1.54E-04 | -1.52E-05 |
| TNF-α(NIL) | 2.72E-04 | -3.89E-05 |
| CCL2 (NIL) | 2.75E-04 | -4.11E-05 |
| IL-17 (TB1) | 9.67E-05 | -6.26E-05 |
| IL-2 (MIT) | 1.71E-04 | -6.72E-05 |
| IP-10 (MIT) | 1.17E-04 | -8.02E-05 |
| IFN-γ (TB1) | 2.98E-05 | -9.94E-05 |
| CCL8 (NIL) | 1.57E-04 | -1.23E-04 |
| IL-2 (NIL) | 2.81E-05 | -1.47E-04 |
| IFN-γ (NIL) | -1.07E-04 | -2.00E-04 |
| IL-6 (MIT) | -2.88E-05 | -2.04E-04 |
| CCL4 (TB1) | -1.60E-04 | -2.31E-04 |
| IL-6 (TB1) | -2.34E-04 | -2.99E-04 |
| IFN-γ (MIT) | -2.24E-04 | -3.08E-04 |
| CCL4 (NIL) | -2.26E-04 | -3.13E-04 |
| CCL3 (TB1) | -3.35E-04 | -3.67E-04 |
| IL-6 (NIL) | -3.58E-04 | -4.37E-04 |
| IP-10 (NIL) | -4.74E-04 | -5.87E-04 |

### Supplemental Table 3. Variable importance metrics (VIM) of the full model for LTBI classification with normalized values as the model input.

| Input Variable | Mean VIM | Median VIM |
| --- | --- | --- |
| IP-10 (TB1-NIL) | 8.88E-02 | 8.82E-02 |
| CCL8 (TB1-NIL) | 1.67E-02 | 1.52E-02 |
| IP-10 (TB1-MIT) | 1.08E-02 | 8.96E-03 |
| IL-2 (TB1-NIL) | 1.01E-02 | 7.78E-03 |
| CCL8 (TB1-MIT) | 8.39E-03 | 6.88E-03 |
| IL-10 (TB1-NIL) | 4.08E-03 | 3.21E-03 |
| IL-10 (TB1-MIT) | 3.00E-03 | 2.43E-03 |
| IL-10 (MIT-NIL) | 2.70E-03 | 2.09E-03 |
| IL-2 (TB1-MIT) | 1.39E-03 | 1.05E-03 |
| CCL4 (TB1-MIT) | 1.12E-03 | 9.33E-04 |
| CCL4 (MIT-NIL) | 1.04E-03 | 7.96E-04 |
| CCL2 (TB1-MIT) | 1.11E-03 | 6.71E-04 |
| IL-15 (MIT-NIL) | 9.37E-04 | 6.64E-04 |
| TNF-α(TB1-MIT) | 7.31E-04 | 5.49E-04 |
| IL-15 (TB1-NIL) | 6.51E-04 | 5.06E-04 |
| IFN-γ (TB1-MIT) | 5.08E-04 | 3.39E-04 |
| TNF-α(MIT-NIL) | 5.24E-04 | 3.32E-04 |
| IFN-γ (MIT-NIL) | 4.88E-04 | 2.59E-04 |
| IP-10 (MIT-NIL) | 4.16E-04 | 2.46E-04 |
| TNF-α(TB1-NIL) | 5.66E-04 | 2.23E-04 |
| IL-2 (MIT-NIL) | 3.60E-04 | 2.14E-04 |
| CCL3 (TB1-NIL) | 8.74E-04 | 1.88E-04 |
| IL-15 (TB1-MIT) | 2.62E-04 | 1.20E-04 |
| CCL3 (MIT-NIL) | 1.18E-04 | 6.16E-05 |
| CCL2 (MIT-NIL) | 2.27E-04 | 1.85E-05 |
| CCL3 (TB1-MIT) | 7.37E-05 | -7.80E-06 |
| IL-17 (MIT-NIL) | 2.12E-04 | -1.60E-05 |
| IL-17 (TB1-NIL) | -6.90E-05 | -1.23E-04 |
| IL-1β (MIT-NIL) | -6.99E-05 | -1.66E-04 |
| IL-6 (TB1-MIT) | -9.75E-05 | -1.72E-04 |
| IL-6 (MIT-NIL) | -9.06E-05 | -1.76E-04 |
| IL-17 (TB1-MIT) | -8.56E-05 | -2.05E-04 |
| IL-1β (TB1-MIT) | -1.17E-04 | -2.24E-04 |
| CCL2 (TB1-NIL) | -1.54E-04 | -2.49E-04 |
| IFN-γ (TB1-NIL) | -2.47E-04 | -3.01E-04 |
| CCL4 (TB1-NIL) | -2.61E-04 | -3.22E-04 |
| CCL8 (MIT-NIL) | -2.95E-04 | -3.36E-04 |
| IL-1β (TB1-NIL) | -3.04E-04 | -3.95E-04 |
| IL-6 (TB1-NIL) | -6.72E-04 | -8.18E-04 |

Supplemental Table 4. Median values of absolute and normalized cytokine levels between LTBI negative and LTBI positive patients. Wilcoxon-Mann-Whitney tests were completed to test significant differences between clinical population distributions. ns p>0.05, * p$\leq$ 0.05, ** p $\leq$ 0.01, *** p $\leq$ 0.001, **** p $\leq$ 0.0001

| Target | Condition | Median concentration (pg/mL) of LTBI– cohort | Median concentration (pg/mL) of LTBI+ cohort | p-value | Significance |
| --- | --- | --- | --- | --- | --- |
| CCL2 | TB1 | 7014.0 | 8459.6 | 0.11 | ns |
| CCL3 | TB1 | 933.6 | 1127.1 | 0.77 | ns |
| CCL4 | TB1 | 2975.0 | 4267.7 | 0.33 | ns |
| CCL8 | TB1 | 72.0 | 342.5 | 1.30E-06 | **** |
| IFN-γ | TB1 | 31.7 | 59.7 | 0.28 | ns |
| IL-10 | TB1 | 852.9 | 65.8 | 0.00011 | *** |
| IL-15 | TB1 | 121.6 | 24.7 | 0.024 | * |
| IL-17 | TB1 | 0.0 | 0.0 | 0.58 | ns |
| IL-1β | TB1 | 710.0 | 630.1 | 0.87 | ns |
| IL-2 | TB1 | 32.8 | 96.9 | 0.00036 | *** |
| IL-6 | TB1 | 1469.7 | 1703.6 | 0.48 | ns |
| IP-10 | TB1 | 210.1 | 922.4 | 1.50E-14 | **** |
| TNF-α | TB1 | 140.7 | 109.2 | 0.44 | ns |
| CCL2 | NIL | 4730.2 | 3109.4 | 0.29 | ns |
| CCL3 | NIL | 1115.9 | 1207.5 | 0.52 | ns |
| CCL4 | NIL | 3421.1 | 3362.2 | 0.64 | ns |
| CCL8 | NIL | 66.9 | 7.0 | 0.11 | ns |
| IFN-γ | NIL | 25.4 | 11.8 | 0.85 | ns |
| IL-10 | NIL | 765.4 | 117.0 | 2.00E-04 | *** |
| IL-15 | NIL | 99.0 | 29.6 | 0.016 | * |
| IL-17 | NIL | 0.0 | 0.0 | 0.32 | ns |
| IL-1β | NIL | 447.1 | 216.3 | 0.02 | * |
| IL-2 | NIL | 30.4 | 19.7 | 0.073 | ns |
| IL-6 | NIL | 1899.3 | 1965.7 | 0.51 | ns |
| IP-10 | NIL | 140.2 | 189.4 | 0.17 | ns |
| TNF-α | NIL | 135.9 | 79.8 | 0.047 | * |
| CCL2 | MIT | 10677.7 | 9291.0 | 0.18 | ns |
| CCL3 | MIT | 8880.6 | 10231.3 | 0.98 | ns |
| CCL4 | MIT | 54015.0 | 46298.2 | 0.13 | ns |
| CCL8 | MIT | 700.6 | 586.6 | 0.64 | ns |
| IFN-γ | MIT | 403.0 | 698.9 | 0.063 | ns |
| IL-10 | MIT | 5066.2 | 2901.0 | 0.0039 | ** |
| IL-15 | MIT | 159.6 | 34.8 | 0.0058 | ** |
| IL-17 | MIT | 23.4 | 0.0 | 0.29 | ns |
| IL-1β | MIT | 3540.7 | 4153.6 | 0.6 | ns |
| IL-2 | MIT | 506.5 | 508.6 | 0.77 | ns |
| IL-6 | MIT | 17852.2 | 16558.0 | 0.9 | ns |
| IP-10 | MIT | 1527.8 | 1453.8 | 0.89 | ns |
| TNF-α | MIT | 529.1 | 510.8 | 0.77 | ns |
| CCL2 | TB1-NIL | 2378.4 | 4211.7 | 0.029 | * |
| CCL3 | TB1-NIL | 23.1 | -234.9 | 0.14 | ns |
| CCL4 | TB1-NIL | 0.0 | 53.6 | 0.032 | * |
| CCL8 | TB1-NIL | 10.8 | 260.0 | 3.80E-08 | **** |
| IFN-γ | TB1-NIL | 0.3 | 5.0 | 0.36 | ns |
| IL-10 | TB1-NIL | 5.5 | 0.0 | 0.84 | ns |
| IL-15 | TB1-NIL | 0.0 | 0.0 | 0.25 | ns |
| IL-17 | TB1-NIL | 0.0 | 0.0 | 0.33 | ns |
| IL-1β | TB1-NIL | 21.5 | 156.5 | 0.08 | ns |
| IL-2 | TB1-NIL | 0.0 | 54.5 | 4.00E-07 | **** |
| IL-6 | TB1-NIL | -24.9 | -72.4 | 0.45 | ns |
| IP-10 | TB1-NIL | 7.4 | 603.8 | 8.30E-15 | **** |
| TNF-α | TB1-NIL | 0.0 | 0.0 | 0.54 | ns |
| CCL2 | TB1-MIT | -3548.0 | 1889.8 | 0.0037 | ** |
| CCL3 | TB1-MIT | -7576.6 | -6344.9 | 0.47 | ns |
| CCL4 | TB1-MIT | -44088.8 | -36428.4 | 0.023 | * |
| CCL8 | TB1-MIT | -513.2 | -182.9 | 0.0088 | ** |
| IFN-γ | TB1-MIT | -304.0 | -478.0 | 0.12 | ns |
| IL-10 | TB1-MIT | -2565.2 | -1870.9 | 0.018 | * |
| IL-15 | TB1-MIT | -34.6 | -7.4 | 0.065 | ns |
| IL-17 | TB1-MIT | 0.0 | 0.0 | 0.19 | ns |
| IL-1β | TB1-MIT | -2720.1 | -3751.4 | 0.97 | ns |
| IL-2 | TB1-MIT | -414.5 | -232.2 | 0.17 | ns |
| IL-6 | TB1-MIT | -15346.9 | -12280.0 | 0.4 | ns |
| IP-10 | TB1-MIT | -1128.2 | -274.3 | 3.70E-06 | **** |
| TNF-α | TB1-MIT | -282.2 | -271.1 | 0.83 | ns |
| CCL2 | MIT-NIL | 5720.6 | 2746.8 | 0.092 | ns |
| CCL3 | MIT-NIL | 7531.9 | 6568.7 | 0.39 | ns |
| CCL4 | MIT-NIL | 44255.6 | 41559.4 | 0.12 | ns |
| CCL8 | MIT-NIL | 575.5 | 537.8 | 0.82 | ns |
| IFN-γ | MIT-NIL | 288.8 | 396.6 | 0.051 | ns |
| IL-10 | MIT-NIL | 2875.1 | 1974.6 | 0.025 | * |
| IL-15 | MIT-NIL | 34.0 | 0.0 | 0.035 | * |
| IL-17 | MIT-NIL | 0.0 | 0.0 | 0.59 | ns |
| IL-1β | MIT-NIL | 2741.9 | 3316.5 | 0.5 | ns |
| IL-2 | MIT-NIL | 424.0 | 307.7 | 0.73 | ns |
| IL-6 | MIT-NIL | 15470.1 | 11504.7 | 0.49 | ns |
| IP-10 | MIT-NIL | 1234.9 | 1129.1 | 0.53 | ns |
| TNF-α | MIT-NIL | 322.3 | 292.8 | 0.82 | ns |

### Supplemental Table 5.Variable importance metrics (VIM) of the full model using absolute cytokine values for classification of high-risk patients from all others in the LTBI+ cohort.

| Input Variable | Mean VIM | Median VIM |
| --- | --- | --- |
| IL-10 (NIL) | 4.22E-02 | 3.31E-02 |
| IL-10 (TB1) | 1.87E-02 | 1.17E-02 |
| IL-6 (NIL) | 5.78E-03 | 3.51E-03 |
| IL-2 (TB1) | 4.97E-03 | 2.53E-03 |
| IL-10 (MIT) | 2.99E-03 | 1.59E-03 |
| TNF-α (TB1) | 3.94E-03 | 1.16E-03 |
| IL-2 (MIT) | 7.92E-04 | 3.15E-04 |
| CCL3 (MIT) | 9.31E-04 | 1.88E-04 |
| CCL8 (NIL) | 3.00E-03 | 1.37E-04 |
| CCL4 (MIT) | 5.19E-04 | -7.53E-05 |
| IL-15 (MIT) | 5.29E-04 | -1.07E-04 |
| TNF-α (NIL) | 5.07E-04 | -2.32E-04 |
| CCL8 (TB1) | 4.57E-04 | -2.54E-04 |
| IL-15 (TB1) | -1.67E-04 | -2.69E-04 |
| IL-6 (TB1) | 1.88E-04 | -3.06E-04 |
| IP-10 (NIL) | 2.23E-05 | -3.74E-04 |
| CCL4 (NIL) | -1.31E-04 | -4.01E-04 |
| TNF-α (MIT) | -1.83E-04 | -4.11E-04 |
| IFN-γ (NIL) | 5.66E-04 | -4.28E-04 |
| CCL4 (TB1) | -3.35E-04 | -4.29E-04 |
| IFN-γ (TB1) | -2.99E-04 | -4.55E-04 |
| IL-15 (NIL) | -1.56E-04 | -4.81E-04 |
| CCL2 (NIL) | 1.51E-04 | -4.95E-04 |
| IL-17 (MIT) | 1.52E-03 | -5.12E-04 |
| IL-1β (TB1) | -2.20E-04 | -5.19E-04 |
| IL-2 (NIL) | -1.81E-04 | -5.45E-04 |
| IFN-γ (MIT) | -5.43E-04 | -5.57E-04 |
| IL-1β (NIL) | 1.89E-04 | -5.65E-04 |
| CCL2 (TB1) | -4.80E-04 | -5.67E-04 |
| IL-17 (TB1) | -4.20E-04 | -5.81E-04 |
| IL-1β (MIT) | -5.58E-04 | -6.19E-04 |
| IL-6 (MIT) | -6.87E-04 | -6.34E-04 |
| CCL3 (TB1) | -6.97E-04 | -6.46E-04 |
| CCL8 (MIT) | -6.28E-04 | -6.53E-04 |
| CCL3 (NIL) | -4.35E-04 | -6.57E-04 |
| IP-10 (TB1) | -6.30E-04 | -6.59E-04 |
| CCL2 (MIT) | -4.56E-04 | -6.95E-04 |
| IP-10 (MIT) | -4.06E-04 | -7.27E-04 |
| IL-17 (NIL) | -9.75E-04 | -9.35E-04 |

### Supplemental Table 6. Variable importance metrics (VIM) of the full model using normalized values for classification of high-risk patients from others in the LTBI+ cohort.

| Input Variable | Mean VIM | Median VIM |
| --- | --- | --- |
| CCL3 (TB1-MIT) | 4.64E-03 | 3.19E-03 |
| IL-10 (MIT-NIL) | 4.59E-03 | 3.16E-03 |
| IL-2 (TB1-NIL) | 4.03E-03 | 2.36E-03 |
| IL-17 (MIT-NIL) | 4.97E-03 | 1.79E-03 |
| IL-10 (TB1-MIT) | 2.67E-03 | 1.33E-03 |
| CCL4 (TB1-MIT) | 2.25E-03 | 1.11E-03 |
| IL-10 (TB1-NIL) | 1.76E-03 | 9.38E-04 |
| CCL3 (MIT-NIL) | 2.09E-03 | 7.96E-04 |
| CCL4 (MIT-NIL) | 1.55E-03 | 5.93E-04 |
| IL-15 (TB1-MIT) | 1.23E-03 | 2.84E-04 |
| IL-15 (MIT-NIL) | 5.00E-04 | 1.01E-04 |
| CCL3 (TB1-NIL) | 1.01E-03 | -3.75E-05 |
| IL-1β (TB1-NIL) | 5.92E-04 | -1.24E-04 |
| IL-2 (MIT-NIL) | 1.67E-04 | -3.47E-04 |
| CCL2 (TB1-NIL) | -3.26E-05 | -3.48E-04 |
| TNF-α (MIT-NIL) | -1.10E-05 | -3.63E-04 |
| IL-1β (TB1-MIT) | 5.59E-04 | -3.84E-04 |
| TNF-α (TB1-MIT) | -1.77E-04 | -4.06E-04 |
| IL-6 (TB1-NIL) | -2.26E-04 | -4.54E-04 |
| TNF-α (TB1-NIL) | 3.83E-04 | -4.86E-04 |
| IFN-γ (TB1-NIL) | -5.65E-05 | -5.33E-04 |
| IL-15 (TB1-NIL) | -3.40E-04 | -5.58E-04 |
| IL-17 (TB1-MIT) | -5.02E-04 | -5.58E-04 |
| CCL8 (MIT-NIL) | -2.96E-04 | -5.81E-04 |
| CCL2 (TB1-MIT) | -4.02E-04 | -6.05E-04 |
| IP-10 (TB1-MIT) | -1.20E-05 | -6.22E-04 |
| CCL8 (TB1-NIL) | -1.79E-04 | -6.32E-04 |
| CCL2 (MIT-NIL) | -2.91E-04 | -6.43E-04 |
| IL-2 (TB1-MIT) | -6.29E-04 | -6.69E-04 |
| IFN-γ (TB1-MIT) | -5.27E-04 | -7.08E-04 |
| IFN-γ (MIT-NIL) | -5.47E-04 | -7.34E-04 |
| IL-17 (TB1-NIL) | -6.12E-04 | -7.78E-04 |
| IL-6 (MIT-NIL) | -7.24E-04 | -8.35E-04 |
| IL-6 (TB1-MIT) | -8.46E-04 | -9.04E-04 |
| IP-10 (TB1-NIL) | -6.75E-04 | -9.40E-04 |
| IP-10 (MIT-NIL) | -7.89E-04 | -9.43E-04 |
| CCL4 (TB1-NIL) | -6.57E-04 | -1.02E-03 |
| IL-1β (MIT-NIL) | -9.11E-04 | -1.03E-03 |
| CCL8 (TB1-MIT) | -9.19E-04 | -1.04E-03 |

### Supplemental Table 7. Variable importance metrics (VIM) of the full model using absolute cytokine values for classification of low-risk patients from all others in the LTBI+ cohort.

| Input Variable | Mean VIM | Median VIM |
| --- | --- | --- |
| IL-2 (TB1) | 1.09E-02 | 7.98E-03 |
| IL-10 (NIL) | 9.02E-03 | 3.72E-03 |
| IL-10 (MIT) | 4.03E-03 | 2.57E-03 |
| CCL8 (TB1) | 4.24E-03 | 2.29E-03 |
| IP-10 (TB1) | 3.65E-03 | 1.51E-03 |
| IL-2 (MIT) | 2.31E-03 | 1.20E-03 |
| TNF-α (TB1) | 2.80E-03 | 8.19E-04 |
| IL-10 (TB1) | 3.15E-03 | 5.26E-04 |
| IL-6 (NIL) | 1.16E-03 | 2.16E-04 |
| CCL4 (TB1) | 4.61E-04 | 1.49E-04 |
| IL-1β (TB1) | 4.01E-04 | 9.26E-05 |
| CCL8 (NIL) | 2.11E-03 | 7.12E-05 |
| IFN-γ (NIL) | 5.75E-04 | -9.11E-05 |
| CCL3 (TB1) | 1.11E-04 | -1.34E-04 |
| IL-17 (TB1) | 2.21E-04 | -2.23E-04 |
| IL-1β (NIL) | 1.16E-03 | -2.60E-04 |
| IL-1β (MIT) | -2.92E-05 | -2.82E-04 |
| TNF-α (MIT) | -1.77E-04 | -3.92E-04 |
| IFN-γ (TB1) | -2.37E-04 | -4.70E-04 |
| CCL3 (MIT) | -1.02E-04 | -4.73E-04 |
| IL-15 (NIL) | -1.98E-04 | -4.80E-04 |
| IFN-γ (MIT) | -4.14E-04 | -5.04E-04 |
| CCL2 (MIT) | -3.10E-04 | -5.20E-04 |
| CCL4 (NIL) | -3.21E-04 | -5.78E-04 |
| CCL3 (NIL) | -4.23E-04 | -5.82E-04 |
| TNF-α (NIL) | -2.61E-04 | -5.86E-04 |
| IL-15 (MIT) | -5.37E-04 | -6.08E-04 |
| IP-10 (MIT) | -1.95E-04 | -6.99E-04 |
| IL-17 (MIT) | 2.24E-04 | -7.88E-04 |
| IL-15 (TB1) | -8.00E-04 | -8.01E-04 |
| IL-6 (TB1) | -8.01E-04 | -8.21E-04 |
| CCL8 (MIT) | -8.19E-04 | -8.52E-04 |
| CCL2 (NIL) | -6.65E-04 | -8.74E-04 |
| IL-6 (MIT) | -8.14E-04 | -8.91E-04 |
| IL-2 (NIL) | -7.59E-04 | -8.96E-04 |
| CCL2 (TB1) | -7.74E-04 | -9.22E-04 |
| CCL4 (MIT) | -9.30E-04 | -1.07E-03 |
| IL-17 (NIL) | -1.25E-03 | -1.20E-03 |
| IP-10 (NIL) | -1.29E-03 | -1.31E-03 |

### Supplemental Table 8. Variable importance metrics (VIM) of the full model using normalized values for classification of low-risk patients from others in the LTBI+ cohort.

| Input Variable | Mean VIM | Median VIM |
| --- | --- | --- |
| IL-2 (TB1-NIL) | 1.04E-02 | 7.47E-03 |
| IL-10 (MIT-NIL) | 3.03E-03 | 1.71E-03 |
| IL-10 (TB1-MIT) | 1.95E-03 | 8.61E-04 |
| IL-1β (TB1-MIT) | 2.46E-03 | 6.91E-04 |
| CCL8 (TB1-NIL) | 2.09E-03 | 6.55E-04 |
| CCL3 (TB1-MIT) | 1.62E-03 | 4.04E-04 |
| IL-1β (TB1-NIL) | 8.86E-04 | 3.78E-04 |
| IL-2 (MIT-NIL) | 1.35E-03 | 2.45E-04 |
| IL-17 (MIT-NIL) | 2.73E-03 | 2.04E-04 |
| IFN-γ (MIT-NIL) | 5.97E-04 | 1.26E-04 |
| TNF-α (TB1-NIL) | 1.34E-03 | -1.56E-04 |
| CCL3 (MIT-NIL) | 4.47E-04 | -2.15E-04 |
| TNF-α (TB1-MIT) | -8.12E-05 | -2.60E-04 |
| IL-6 (TB1-NIL) | 3.68E-06 | -2.89E-04 |
| IFN-γ (TB1-MIT) | 4.68E-05 | -3.11E-04 |
| IL-17 (TB1-MIT) | -2.10E-04 | -3.26E-04 |
| CCL2 (TB1-MIT) | -5.50E-05 | -3.38E-04 |
| CCL2 (MIT-NIL) | -2.74E-04 | -4.27E-04 |
| IL-6 (MIT-NIL) | -4.47E-04 | -4.34E-04 |
| TNF-α (MIT-NIL) | -1.01E-04 | -4.78E-04 |
| IL-17 (TB1-NIL) | -3.47E-04 | -4.81E-04 |
| IL-15 (TB1-MIT) | -3.10E-04 | -5.89E-04 |
| CCL2 (TB1-NIL) | -3.06E-04 | -6.01E-04 |
| IP-10 (TB1-NIL) | 7.52E-05 | -6.16E-04 |
| IL-15 (MIT-NIL) | -5.65E-04 | -6.83E-04 |
| IL-6 (TB1-MIT) | -6.53E-04 | -6.88E-04 |
| CCL3 (TB1-NIL) | -3.18E-04 | -7.24E-04 |
| CCL8 (MIT-NIL) | -6.59E-04 | -7.57E-04 |
| IL-10 (TB1-NIL) | -5.66E-04 | -7.82E-04 |
| CCL8 (TB1-MIT) | -5.79E-04 | -8.38E-04 |
| IL-2 (TB1-MIT) | -7.93E-04 | -8.76E-04 |
| IL-1β (MIT-NIL) | -8.48E-04 | -9.11E-04 |
| IP-10 (TB1-MIT) | -7.79E-04 | -9.65E-04 |
| CCL4 (TB1-MIT) | -6.32E-04 | -9.84E-04 |
| IP-10 (MIT-NIL) | -9.22E-04 | -1.07E-03 |
| CCL4 (TB1-NIL) | -8.66E-04 | -1.07E-03 |
| IFN-γ (TB1-NIL) | -8.74E-04 | -1.09E-03 |
| IL-15 (TB1-NIL) | -1.11E-03 | -1.17E-03 |
| CCL4 (MIT-NIL) | -9.89E-04 | -1.27E-03 |

Supplemental Table 9. Median values of absolute and normalized cytokine levels between low-risk and high-risk designated patients within the LTBI+ patient population**.** Patients without either high or low risk designations were excluded from analysis. Wilcoxon-Mann-Whitney tests were completed to test significant differences between clinical population distributions. ns < 0.05, * p$\leq$ 0.05, ** p $\leq$ 0.01, *** p $\leq$ 0.001, **** p $\leq$ 0.0001

| Target | Condition | Median concentration (pg/mL) of low-risk cohort | Median concentration (pg/mL) of high-risk cohort | p-value | Significance |
| --- | --- | --- | --- | --- | --- |
| CCL2 | TB1 | 7276.1 | 8313.6 | 0.42 | ns |
| CCL3 | TB1 | 1074.4 | 1090.1 | 0.63 | ns |
| CCL4 | TB1 | 3999.7 | 4730.6 | 0.5 | ns |
| CCL8 | TB1 | 151.7 | 421.6 | 0.011 | * |
| IFN-γ | TB1 | 60.3 | 53.4 | 0.6 | ns |
| IL-10 | TB1 | 0.0 | 209.4 | 0.0018 | ** |
| IL-15 | TB1 | 19.8 | 43.4 | 0.17 | ns |
| IL-17 | TB1 | 0.0 | 0.0 | 0.44 | ns |
| IL-1β | TB1 | 366.9 | 719.9 | 0.13 | ns |
| IL-2 | TB1 | 33.6 | 139.7 | 0.0014 | ** |
| IL-6 | TB1 | 1367.4 | 1951.9 | 0.24 | ns |
| IP-10 | TB1 | 659.6 | 1053.5 | 0.036 | * |
| TNF-α | TB1 | 25.0 | 173.3 | 0.011 | * |
| CCL2 | NIL | 2463.0 | 3887.3 | 0.24 | ns |
| CCL3 | NIL | 1506.6 | 1173.3 | 0.81 | ns |
| CCL4 | NIL | 2672.4 | 3533.2 | 0.47 | ns |
| CCL8 | NIL | 0.0 | 36.7 | 0.038 | * |
| IFN-γ | NIL | 26.5 | 5.2 | 0.11 | ns |
| IL-10 | NIL | 0.0 | 187.2 | 0.00029 | *** |
| IL-15 | NIL | 20.4 | 48.1 | 0.19 | ns |
| IL-17 | NIL | 0.0 | 0.0 | 0.77 | ns |
| IL-1β | NIL | 104.5 | 313.5 | 0.051 | ns |
| IL-2 | NIL | 11.9 | 26.2 | 0.074 | ns |
| IL-6 | NIL | 1182.5 | 2380.2 | 0.1 | ns |
| IP-10 | NIL | 188.0 | 285.2 | 0.46 | ns |
| TNF-α | NIL | 28.5 | 97.2 | 0.033 | * |
| CCL2 | MIT | 7224.7 | 9768.9 | 0.7 | ns |
| CCL3 | MIT | 12914.1 | 8325.2 | 0.067 | ns |
| CCL4 | MIT | 46578.8 | 43792.7 | 0.96 | ns |
| CCL8 | MIT | 535.5 | 863.8 | 0.93 | ns |
| IFN-γ | MIT | 617.5 | 698.9 | 0.6 | ns |
| IL-10 | MIT | 1189.6 | 5023.4 | 0.0012 | ** |
| IL-15 | MIT | 37.3 | 48.1 | 0.32 | ns |
| IL-17 | MIT | 0.0 | 8.7 | 0.24 | ns |
| IL-1β | MIT | 2455.9 | 4361.7 | 0.26 | ns |
| IL-2 | MIT | 238.3 | 590.3 | 0.028 | * |
| IL-6 | MIT | 15372.0 | 16649.6 | 0.91 | ns |
| IP-10 | MIT | 1001.3 | 2223.2 | 0.29 | ns |
| TNF-α | MIT | 417.7 | 649.9 | 0.19 | ns |
| CCL2 | TB1-NIL | 4750.0 | 3478.4 | 0.84 | ns |
| CCL3 | TB1-NIL | -290.3 | -77.9 | 0.41 | ns |
| CCL4 | TB1-NIL | 360.3 | 0.0 | 0.93 | ns |
| CCL8 | TB1-NIL | 111.2 | 391.5 | 0.084 | ns |
| IFN-γ | TB1-NIL | 8.6 | 2.8 | 0.76 | ns |
| IL-10 | TB1-NIL | 0.0 | 0.0 | 0.46 | ns |
| IL-15 | TB1-NIL | 0.0 | 0.0 | 0.83 | ns |
| IL-17 | TB1-NIL | 0.0 | 0.0 | 0.6 | ns |
| IL-1β | TB1-NIL | 161.1 | 73.7 | 0.64 | ns |
| IL-2 | TB1-NIL | 29.3 | 62.1 | 0.013 | * |
| IL-6 | TB1-NIL | 5.8 | -104.9 | 0.78 | ns |
| IP-10 | TB1-NIL | 503.4 | 684.5 | 0.12 | ns |
| TNF-α | TB1-NIL | 0.0 | 20.7 | 0.49 | ns |
| CCL2 | TB1-MIT | 1594.7 | 2170.7 | 0.38 | ns |
| CCL3 | TB1-MIT | -8811.1 | -5893.6 | 0.073 | ns |
| CCL4 | TB1-MIT | -35933.7 | -36428.4 | 0.83 | ns |
| CCL8 | TB1-MIT | -371.6 | -110.7 | 0.38 | ns |
| IFN-γ | TB1-MIT | -304.2 | -605.6 | 0.1 | ns |
| IL-10 | TB1-MIT | -843.1 | -2936.7 | 0.0061 | ** |
| IL-15 | TB1-MIT | -3.7 | -10.6 | 0.17 | ns |
| IL-17 | TB1-MIT | 0.0 | 0.0 | 0.3 | ns |
| IL-1β | TB1-MIT | -1621.5 | -3839.7 | 0.15 | ns |
| IL-2 | TB1-MIT | -94.9 | -240.2 | 0.16 | ns |
| IL-6 | TB1-MIT | -12629.1 | -12054.7 | 0.58 | ns |
| IP-10 | TB1-MIT | -407.7 | -332.3 | 0.68 | ns |
| TNF-α | TB1-MIT | -154.2 | -324.6 | 0.17 | ns |
| CCL2 | MIT-NIL | 2051.6 | 3073.4 | 0.72 | ns |
| CCL3 | MIT-NIL | 8942.1 | 5826.4 | 0.14 | ns |
| CCL4 | MIT-NIL | 40636.9 | 40823.8 | 0.76 | ns |
| CCL8 | MIT-NIL | 530.9 | 572.5 | 0.91 | ns |
| IFN-γ | MIT-NIL | 300.1 | 459.1 | 0.11 | ns |
| IL-10 | MIT-NIL | 925.2 | 3792.8 | 0.0041 | ** |
| IL-15 | MIT-NIL | 0.0 | 0.0 | 0.34 | ns |
| IL-17 | MIT-NIL | 0.0 | 0.0 | 0.3 | ns |
| IL-1β | MIT-NIL | 2418.9 | 3646.9 | 0.35 | ns |
| IL-2 | MIT-NIL | 166.4 | 503.3 | 0.0099 | ** |
| IL-6 | MIT-NIL | 10889.2 | 12285.3 | 0.63 | ns |
| IP-10 | MIT-NIL | 846.8 | 1129.1 | 0.65 | ns |
| TNF-α | MIT-NIL | 192.9 | 359.2 | 0.18 | ns |

## Supporting Information References

1. Yu D, Humar M, Meserve K, Bailey RC, Chormaic SN, Vollmer F. Whispering-gallery-mode sensors for biological and physical sensing. Nat Rev Methods Primer. 2021;1: 83. doi:10.1038/s43586-021-00079-2

2. Iqbal M, Gleeson MA, Spaugh B, Tybor F, Gunn WG, Hochberg M, et al. Label-Free Biosensor Arrays Based on Silicon Ring Resonators and High-Speed Optical Scanning Instrumentation. IEEE J Sel Top Quantum Electron. 2010;16: 654–661. doi:10.1109/JSTQE.2009.2032510

3. Bailey RC, Washburn AL, Qavi AJ, Iqbal M, Gleeson M, Tybor F, et al. A robust silicon photonic platform for multiparameter biological analysis. In: Kubby JA, Reed GT, editors. San Jose, CA; 2009. p. 72200N. doi:10.1117/12.809819

4. Robison HM, Bailey RC. A Guide to Quantitative Biomarker Assay Development using Whispering Gallery Mode Biosensors: Whispering Gallery Mode Biosensors. Curr Protoc Chem Biol. 2017;9: 158–173. doi:10.1002/cpch.23

5. Meserve K, Qavi AJ, Aman MJ, Vu H, Zeitlin L, Dye JM, et al. Detection of biomarkers for filoviral infection with a silicon photonic resonator platform. STAR Protoc. 2022;3: 101719. doi:10.1016/j.xpro.2022.101719

6. Move Data, not Blood - 30 Minutes Results - Onsite Lab Automation. 26 Apr 2021 [cited 19 Feb 2024]. Available: https://www.genalyte.com/

7. Zhu R, Zeng D, Kosorok MR. Reinforcement Learning Trees. J Am Stat Assoc. 2015;110: 1770–1784. doi:10.1080/01621459.2015.1036994

8. Breiman L. Random Forests. Mach Learn. 2001;45: 5–32. doi:10.1023/A:1010933404324

9. Wu L. Mixed Effects Models for Complex Data. CRC Press/A Chapman & Hall Book; 2010.

10. Gałecki A, Burzykowski T. Linear Mixed-Effects Model. In: Gałecki A, Burzykowski T, editors. Linear Mixed-Effects Models Using R: A Step-by-Step Approach. New York, NY: Springer; 2013. pp. 245–273. doi:10.1007/978-1-4614-3900-4_13

11. Zeger SL, Liang K-Y, Albert PS. Models for Longitudinal Data: A Generalized Estimating Equation Approach. Biometrics. 1988;44: 1049–1060. doi:10.2307/2531734

12. Muthén B, Asparouhov T. Multilevel Regression Mixture Analysis. J R Stat Soc Ser A Stat Soc. 2009;172: 639–657. doi:10.1111/j.1467-985X.2009.00589.x

13. Ishwaran H, Lu M. Standard errors and confidence intervals for variable importance in random forest regression, classification, and survival. Stat Med. 2019;38: 558–582. doi:10.1002/sim.7803

14. Ishwaran H, Kogalur UB, Gorodeski EZ, Minn AJ, Lauer MS. High-Dimensional Variable Selection for Survival Data. J Am Stat Assoc. 2010;105: 205–217.
